# Trellis systems ameliorate heat damage by regulating canopy temperature, photosynthetic efficiency and leaf microstructure of grapevine

**DOI:** 10.3389/fpls.2025.1648999

**Published:** 2026-02-04

**Authors:** Ling Luo, Xinya Liu, Xiulan Lyu, Qi Zhong, Yijun Ma, Ran Li, Wei Liu

**Affiliations:** 1 Institute of Horticulture, Sichuan Academy of Agricultural Sciences, Chengdu, China; 2 College of Horticulture, Sichuan Agricultural University, Chengdu, China; 3 Sichuan Key Laboratory for Germplasm Innovation and Utilization of Horticultural Crops, Chengdu, China; 4 Key Laboratory of Horticultural Crop Biology and Germplasm Creation in Southwest China, Ministry of Agriculture and Rural Affairs, Chengdu, China

**Keywords:** heat damage, trellis systems, ‘shine muscat’, photosynthesis, stomata, chloroplasts

## Abstract

**Introduction:**

Global warming has made heat stress a major constraint on grapevine growth and grape production. This study aimed to evaluate the heat tolerance of three prevalent trellis systems—V-shaped (VT), upward-trained pendulous (U-PT), and H-shaped (HT)—for *Vitis labruscana* × *V. vinifera* ‘Shine Muscat’. We specifically tested the hypothesis that U-PT enhances heat tolerance by optimizing canopy structure to mitigate high-temperature stress, thereby alleviating its negative impacts on stomatal function, chloroplast integrity, and photosynthetic performance.

**Methods:**

Under summer rain-shelter cultivation, the three trellis systems were compared using five-year-old ‘Shine Muscat’ grapevines based on canopy temperature, relative humidity, leaf sunburn, chlorophyll content, stomatal morphology, chloroplast ultrastructure, leaf gas exchange, and chlorophyll fluorescence.

**Results:**

Under prolonged heat stress, stomatal aperture dimensions and aperture ratio decreased (*p* < 0.05) without significant changes in stomatal density (*p* > 0.05). Chloroplasts displayed volumetric expansion and substantial lipid droplet accumulation, with particularly pronounced chloroplast envelope disintegration in HT. From Day 3 to Day 15 of prolonged high-temperature stress, net photosynthetic rate (*P*_n_), stomatal conductance (*g*_s_), transpiration rate (*T*_r_), intercellular CO₂ concentration (*C*_i_), chlorophyll content, and photochemical quenching coefficient (*qP*) initially increased, peaking on Day 3 or Day 6, then progressively declined. Maximum energy conversion efficiency (*F*_v_/*F*_m_), actual photochemical efficiency (*Φ*_PSII_) and non-photochemical quenching coefficient (*NPQ*) remained stable on Day 3. Subsequently, *F*_v_/*F*_m_ and *Φ*_PSII_ gradually decreased, while *NPQ* gradually increased. Comparative analysis revealed U-PT maintained the lowest intensity and shortest duration of high canopy temperatures along with higher canopy relative humidity, exhibited the minimal leaf sunburn damage index, and sustained the highest stomatal aperture, *P*_n_, *F*_v_/*F*_m_, *Φ*_PSII_, *qP*, and chlorophyll content, and most stable chloroplast structure, whereas HT performed poorest. The principal component analysis (PCA) confirmed U-PT as the most heat-tolerant trellis system.

**Discussion:**

These findings could provide insights into the responses and adaptions of grapevines to heat stress and aid in the optimization of heat-tolerant trellis systems under everchanging climatic conditions.

## 1 Introduction

Extreme heat conditions have increased in frequency and intensity due to global warming, particularly in temperate, subtropical, and tropical countries where summer temperatures frequently surpass 40 °C—posing a significant threat to plant growth and development (Wang et al., 2022; Yu et al., 2022). In viticulture, prolonged exposure to temperatures ≥35 °C induces leaf scorching and curling, while berries develop characteristic thermal necrosis lesions, severely reducing both yield and fruit quality (Dou et al., 2021). Notably, the widespread adoption of rain-shelter cultivation systems in modern vineyards, though effective against meteorological hazards (frost, precipitation, hail, wind) and pathogen outbreaks (Du et al., 2015), inadvertently creates thermal accumulation within vineyard ecosystems, exacerbating heat stress severity (Alonso et al., 2021). Consequently, developing scientifically sound methods to mitigate heat stress has become imperative for sustaining vineyard productivity under climate warming.

Tree form fundamentally governs leaf spatial distribution, thereby critically influencing both plant growth vigor and orchard microenvironmental parameters including light intensity, temperature, humidity, and ventilation (Reynolds and Vanden Heuvel, 2009; Gladstone and Dokoozlian, 2003; Han et al., 2023). Previous studies on Vitis vinifera ‘Cabernet Sauvignon’ have demonstrated that compared with the Four-Arm Kniffin (4AK) trellis system, Single Guyot (SG) and Vertical Shoot-Positioned (VSP) trellis system significantly reduce canopy temperature and humidity while enhancing light penetration (Liu et al., 2015). These findings suggest that trellis system adjustment in viticulture can improve vineyard microclimate and enhance plant stress tolerance, thereby improving grapevine adaptation to high-temperature environments. Compared with replacing heat-tolerant varieties or repeated annual applications of chemical protectants, trellis system adjustment represents a more straightforward and implementable technical measure, particularly when planned during new vineyard establishment.

Photosynthesis constitutes the physiological foundation of crop productivity and quality formation while exhibiting exceptional sensitivity to heat stress (Hussain et al., 2021). High temperatures induce stomatal closure, accelerate chlorophyll degradation, and impair key photosynthetic components including photosystem II complex (PSII), cytochrome b6f complex, and Rubisco activase. These perturbations collectively impair light-harvesting capacity, disrupt electron transport chains, suppress carbon assimilation, and elevate energy dissipation via thermal emission and photorespiration, ultimately compromising photosynthetic efficiency (Zhou et al., 2023; Crafts-Brandner and Salvucci, 2000). Chlorophyll fluorescence analysis serves as a critical phenotyping tool for dissecting photosynthetic performance under abiotic stress (Doğru, 2021). This technique enables non-invasive quantification of light energy partitioning, excitation energy transfer dynamics, and PSII reaction center integrity (Maxwell and Johnson, 2000). Under heat stress, PSII photoinhibition manifests as increased minimum fluorescence (*F*_o_) and non-photochemical quenching coefficient (*NPQ*) (Xue et al., 2010; Sun et al., 2017), concomitant with reductions in maximum quantum yield of PSII (*F*_v_/*F*_m_), electron transport rate (*ETR*), and photochemical quenching coefficient (*qP*) (He et al., 2022; Qin et al., 2010). *F*_v_/*F*_m_ shows a significant positive correlation with intrinsic heat tolerance in plants and has been widely used as a physiological marker for the identification of heat-resistant genotypes (Li et al., 2021).

*Vitis labruscana* × *V. vinifera* ‘Shine Muscat’, a premium table grape cultivar, features a thin pericarp, crisp flesh with a distinctive rose aroma, and soluble solids content exceeding 20%. Its exceptional agronomic traits— including disease resistance, high yield potential, superior transport tolerance, and long shelf life—have established it as a globally predominant commercial variety (Yamada and Sato, 2016; Shirasawa et al., 2022; Yuan et al., 2024). However, the current understanding of how various trellis systems influence heat tolerance mechanisms in ‘Shine Muscat’ remains limited.

This study employed 5-year-old ‘Shine Muscat’ as experimental material under summer rain-shelter cultivation. Three regionally prevalent trellis systems in southern China were evaluated: V-shaped trellis (VT), upward-trained pendulous trellis (U-PT), and H-shaped trellis (HT). By comparative analysis of changes in canopy temperature and multidimensional photosynthetic characteristics (including stomatal morphology, chloroplast ultrastructure, gas exchange parameters, and chlorophyll fluorescence parameters) during high-temperature periods, we systematically elucidate the photosynthetic response mechanisms of fruit-bearing grapevines to summer heat stress and the mechanisms of trellis systems alleviating high-temperature stress. We propose and verify the following scientific hypothesis: U-PT enhances the heat tolerance of grapevines by optimizing canopy structure, which acts first to reduce canopy temperature, thus weakening the vicious cycle of “high-temperature stress - stomatal closure - chloroplast damage - photoinhibition”. The findings will establish a theoretical foundation for selecting heat-tolerant trellis systems and investigating heat tolerance mechanisms in viticulture.

## 2 Materials and methods

### 2.1 Orchard location and plant material

The experiment was conducted in August 2022 at the Tianfu New Area Experimental Station (30°27′N, 104°13′E; 500 m elevation) of the Sichuan Academy of Agricultural Sciences, China. The site experiences a subtropical monsoon climate characterized by humid summers. Based on data from the meteorological station near the experimental site, the mean annual climate condtitions over the past five years (2020–2024) were as follows: air temperature, 16.5 °C; relative humidity, 79%; sunshine duration, 1032.9 hours; and rainfall, 895.6 mm, the above data were retrospectively calculated to characterize the climatic context and were not available during the planning or execution of the experiment. Soil analysis revealed a medium loam texture with pH 6.97, organic matter content of 2.19%, and bulk density of 1.65 g·cm^-3^.

**Figure 1:**
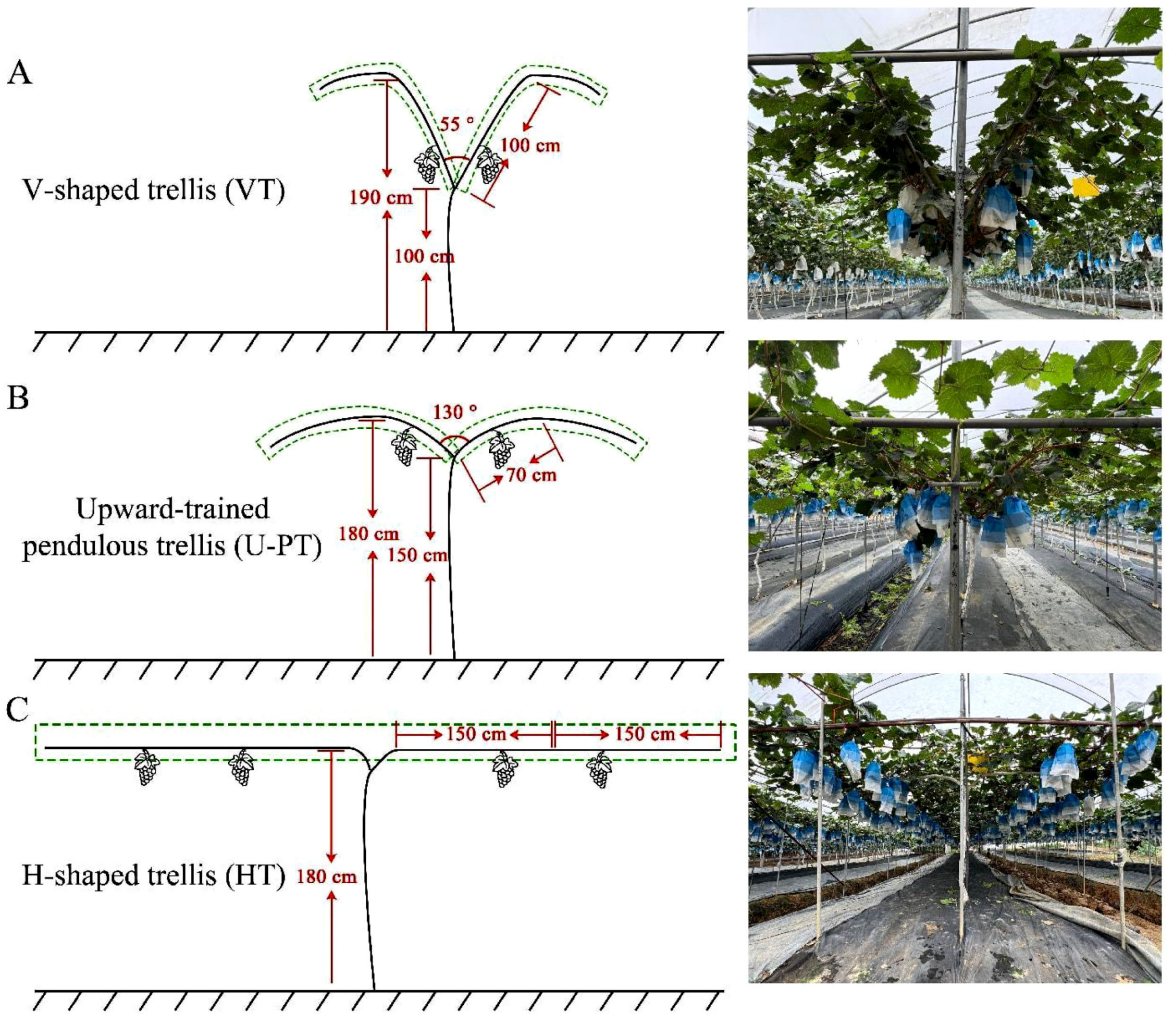
Diagrammatic drawing and field photographs of the three grapevine trellis systems. **(A)** V-shaped trellis (VT); **(B)** Upward-trained pendulous trellis (U-PT); **(C)** H-shaped trellis (HT).

Five-year-old ‘Shine Muscat’ (*Vitis labruscana* Bailey × *V. vinifera* L.) with uniform vigor were selected. Three trellis systems were established:

V-shaped trellis (VT; **Figure 1A**): Bilateral cordons were trained horizontally on wires at 1.0 m height. Newly developed shoots grew upward at 60°from horizontal until reaching 100 cm, then drooped freely. The canopy height was 1.9 m with 3.0 × 2.0 m (row × vine) spacing.
Upward-trained pendulous trellis (U-PT; **Figure 1B**): Bilateral cordons were horizontally fixed on wires at 1.5 m height. Newly developed shoots initially grew upward at 25°from horizontal, transitioning to free pendulous growth at 70 cm length. The canopy height reached 1.8 m with identical 3.0 × 2.0 m spacing.
H-shaped trellis (HT; **Figure 1C**): Newly developed shoots were trained horizontally with 1.8 m canopy height. Spacing was 6.0 × 4.0 m (row × vine).

The trial was conducted following a completely randomized design with three replicates, where each replicate of the three trellis systems (VT, U-PT, HT) was established in an independent rain-shelter structure plastic film roofing and insect-proof mesh sidewalls, totaling nine structures. Each structure spanned 6 m (width) × 125 m (length), with 4.3 m ridge height and 2.5 m gutter height. Vines were planted in north-south oriented rows with 20 cm spacing between shoots on each side. All vines received uniform pruning, flower and fruit management, water-fertilizer regime, and pest and disease control, details are provided in the **Supplementary Materials**.

### 2.2 High-temperature treatment and sampling

**Figure 2:**
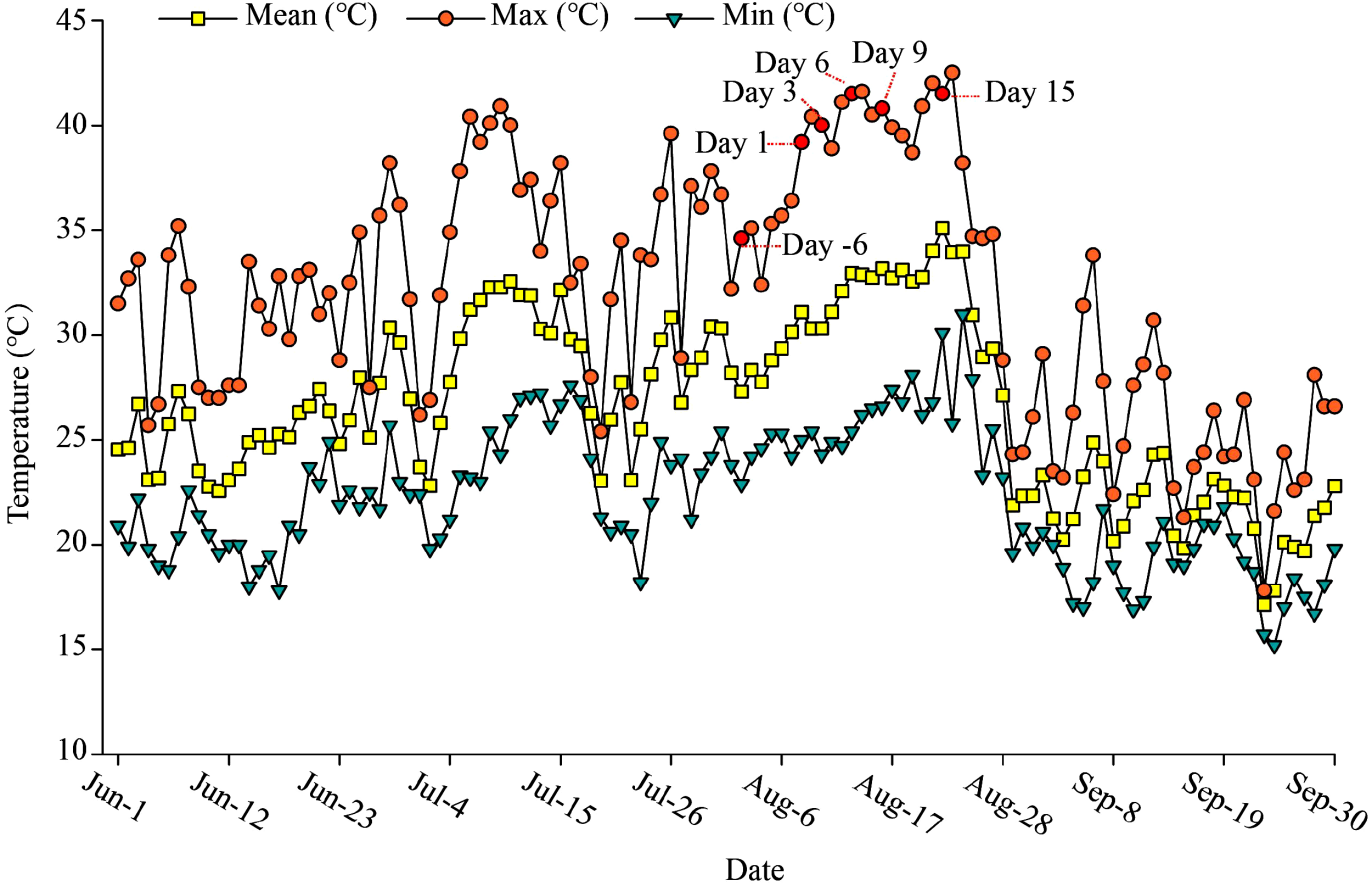
Daily mean, minimum, and maximum air temperatures at the experimental site (June-September). Day -6 denotes the 6^th^ day prior to the onset of the high-temperature period, Day 1, Day 3, Day 6, Day 9 and Day 15 denote the 1^st^, 3^rd^, 6^th^, 9^th^, and 15^th^ days after the start of the high-temperature period, respectively.

The experimental site experiences continuous high-temperature weather every summer. In this study, we define the first day in August with a daily maximum temperature exceeding 38°C as the start of the high-temperature period. On 2 August 2022 (defined as Day -6), the maximum temperature at the experimental site remained below 38°C according to weather forecasts. We therefore defined this day as the pre-stress reference timepoint and collected initial parameter data. On 8 August (defined as Day 1), when temperatures peaked at 39°C with meteorological projections of sustained heatwaves (>38°C), we initiated continuous investigation into plant responses to heat stress. Subsequent measurements were conducted at 3-day intervals (Days 3, 6, 9, and 15; 10, 13, 16, and 22 August respectively) until daily maximum temperatures subsided below 38°C. As shown in **Figure 2**, the pre-Day 1 period (before 8 August) had daily maximum temperatures below 38°C, while the high-temperature period (8–24 August) saw daily temperatures exceeding 38°C for at least 1 hour, with a peak temperature of 42°C.

Before experiment initiation, 10 uniformly growing vines in central rows of each rain-sheltered structure per treatment were tagged as fixed sample plants. The following sampling was performed within each structure: Six leaves at the 7^th^ node of newly developed shoots per treatment were selected for diurnal monitoring of photosynthetic parameters and chlorophyll fluorescence parameters at each designated timepoint (Day -6, 3, 6, 9, 15). During 12:00–14:00 at each designated time point, 10 leaves from the 6^th^-8^th^ nodes per treatment were collected for chlorophyll content determination. Five 7^th^-node leaves collected on Day -6 and Day 15 were analyzed for stomatal morphology and chloroplast ultrastructure. All samples were obtained from the same canopy orientation. Each treatment was replicated three times, meaning the above sampling was identically conducted in three independent structures.

### 2.3 Canopy temperature monitoring

For each trellis system, three representative canopy areas with uniform leaf distribution were selected. ZDR-U1W1S-T2 temperature and relative humidity (RH) loggers (Hangzhou Zheda Instruments Co., Ltd., Hangzhou, China; ± 0.5°C and ±3% RH accuracy) were mounted at the 4^th^-5^th^ nodes of newly developed shoots (near fruit clusters) to record hourly data throughout August 2022 (1-31 August; total 744 hours). We calculated the daily and monthly mean, maximum, and minimum for both canopy temperature and RH. Additionally, we determined the cumulative durations and corresponding proportions (of the total 744-hour period) for the following parameters: temperatures ≥40°C and ≥45°C, RH within 60-80%, and RH >80%.

### 2.4 Leaf sunburn evaluation

Leaf sunburn assessment was conducted on Day 15 according to the method of Li et al. (2011) with slight modifications. Briefly, ten pre-marked plants (constituting three biological replicates) per treatment were used. Six newly developed shoots per plant were randomly sampled to examine all expanded leaves. Sunburn severity was classified using a six-grade system based on thermal necrosis area percentage: Grade 0 (0%), 1 (≥0% and <5%), 2 (≥5% and <10%), 3 (≥10% and <25%), 4 (≥25% and <50%), and 5 (≥50%). Sunburn incidence rate and sunburn damage index were calculated as follows:


$$\text{Sunburn incidence rate}=\frac{\text{affected leaves}}{\text{total assessed leaves}}\times 100$$

$$\text{Sunburn damage index}=\frac{0a+1b+2c+3d+4e+5f}{5n}\times 100$$

where a-f are Grade 0-5 leaf counts, and n is the total number of assessed leaves.

### 2.5 Scanning electron microscopy observation of stomatal morphology

The observations by scanning electron microscopy were conducted by Wuhan Servicebio Technology Co., Ltd. (Wuhan, China), following their standard procedures (Servicebio, 2025; available online: https://www.service-bio.com/data-detail?id=4303&code=DJSYBG). Briefly, leaf segments (5 × 5 mm) adjacent to the primary veins were immediately fixed in 2% paraformaldehyde and 2.5% glutaraldehyde at 4°C. Samples were rinsed three times with 0.1 M phosphate buffer (PB, pH 7.4), post-fixed in 1% osmium tetroxide for 1-2 h, then rinsed three additional times with PB. Dehydration was performed through a graded ethanol series (30%, 50%, 70%, 80%, 90%, 95%, 100%, 100%; 15 min per step). Samples were transitioned through isoamyl acetate for 15 min, followed by critical point drying and gold sputter-coating.

Observations were conducted using an SU8100 field-emission scanning electron microscope (Hitachi, Tokyo, Japan). Stomatal density (number per mm^2^) and aperture ratio (open/total stomata × 100%) were calculated from 200× micrographs. Stomatal dimensions (length, width, aperture length, aperture width) were measured from 2500× micrographs. Quantifications were performed using Adobe Photoshop CC 2023 (version 24.0; Adobe Inc., San Jose, CA). For each treatment, five biological replicates were analyzed with three random fields of view per replicate.

Stomatal length was defined as the maximum distance between the poles of the guard cell pair. Stomatal width represented the maximum dimension perpendicular to the length axis. Aperture length was measured as the longest axis of the stomatal pore, with aperture width defined as the maximum dimension perpendicular to the aperture length axis.

### 2.6 Transmission electron microscopy observation of chloroplast ultrastructure

The observations by transmission electron microscopy were conducted by Wuhan Servicebio Technology Co., Ltd. (Wuhan, China), following their standard procedures (Servicebio, 2025; available online: https://www.service-bio.com/data-detail?id=4300&code=DJSYBG). Briefly, leaf segments (2 × 5 mm) adjacent to the primary veins were immediately fixed in 2% paraformaldehyde and 2.5% glutaraldehyde at 4 °C. Samples were rinsed three times with 0.1 M phosphate buffer (PB, pH 7.4), post-fixed in 1% osmium tetroxide for 7 h, then rinsed three additional times with PB. Dehydration was performed through a graded ethanol series (30%, 50%, 70%, 80%, 95%, 100%, 100%; 1 h per step). Tissues were embedded in Spurr’s resin, transferred to microcentrifuge tubes, and thermally cured in a dry oven at 60 °C for 48 h. Embedded blocks were sectioned into 60-80 nm slices using an EM UC7 ultramicrotome (Leica Microsystems, Wetzlar, Germany). Sections were stained with 2% uranyl acetate for 8 min (protected from light), rinsed with ultrapure water, then counterstained with 2.6% lead citrate for 8 min in a CO_2_-free chamber, followed by ultrapure water rinses. Samples were imaged using an HT7800 transmission electron microscope (TEM) (Hitachi High-Tech Corporation, Tokyo, Japan). Quantifications were performed using Adobe Photoshop CC 2023 (version 24.0; Adobe Inc., San Jose, CA) by measuring the length and width of chloroplasts and lipid droplets and by counting the number of lipid droplets per chloroplast. Five random fields of view per section were analyzed.

### 2.7 Chlorophyll content determination

Chlorophyll content was determined according to the method of He et al. (2018) with slight modifications. Briefly, Leaf samples (100 mg fresh weight, FW) were dark-extracted in 10 mL 80% acetone for 24 h at 25°C. Absorbance measurements at 663 nm (A_663_) and 645 nm (A_645_) wavelengths were conducted using a UV-1800 UV-Vis spectrophotometer (Shimadzu Corporation, Kyoto, Japan). Chlorophyll contents were calculated as follows:


$$\text{Chlorophyll a }(\text{mg}\cdot {\text{g}}^{-1}\text{FW})=\frac{(12.72{\text{A}}_{663}-2.59{\text{A}}_{645})V}{1000W}$$

$$\text{Chlorophyll b }(\text{mg}\cdot {\text{g}}^{-1}\text{FW})=\frac{(22.88{\text{A}}_{645}-4.67{\text{A}}_{663})V}{1000W}$$

Total chlorophyll(mg/g FW)=Chl a+Chl b

Where *V* is the extraction volume (mL), and *W* is fresh weight (g).

### 2.8 Gas exchange measurements

Leaf photosynthetic parameters, including net photosynthetic rate (*P*_n_), stomatal conductance (*g*_s_), transpiration rate (*T*_r_), and intercellular CO_2_ concentration (*C*_i_), were measured at 2-h intervals from 08:00 to 18:00 using an LI-6400XT portable photosynthesis system (LI-COR Biosciences, Lincoln, NE, USA). The measurement procedure followed the method described by Rogiers et al. (2020). The leaf chamber was maintained at: 500 μmol·s^-1^ airflow rate, 60% relative humidity, 400 μmol·mol^-1^ reference CO_2_ concentration, 25°C block temperature, and 1500 μmol·m^-2^·s^-1^ photosynthetic photon flux density (PPFD). The intrinsic water-use efciency (WUEi) was calculated according to Wang et al. (2020): WUEi=*P*_n_/*g*_s_.

### 2.9 Chlorophyll fluorescence analysis

Chlorophyll fluorescence parameters were recorded synchronously with gas exchange measurements using a PAM-2500 portable modulated chlorophyll fluorometer (Walz GmbH, Effeltrich, Germany). The measurement procedure followed the method described by Pollet et al. (2010). After the leaves were dark-adapted for 30 min, the minimum fluorescence (*F*_0_) was first measured with a weak measuring light (~0.1 μmol·m^-2^·s^-1^); Subsequently, a saturating light pulse (>1500 μmol·m^-2^·s^-1^, 0.8 s duration) was applied to measure the maximum fluorescence (*F*_m_). Afterward, the leaves were light-adapted under natural light for 30 minutes. Once the fluorescence signal stabilized, the steady-state fluorescence (*F*_s_) was recorded. Another saturating pulse (>1500 μmol·m^-2^·s^-1^, 0.8 s) was then applied to determine the maximum light-adapted fluorescence (*F*_m_′). The leaf was subsequently shaded for 3 seconds, followed by exposure to far-red light for 5 seconds to measure the minimum light-adapted fluorescence (*F*_0_′). The following parameters were calculated according to Roháček (2002): maximum quantum yield of PSII [*F*_v_/*F*_m_ = (*F*_m_ – *F*_0_)/*F*_m_]; effective quantum yield of PSII [√_PSII_ = (*F*_m_′ – *F*_s_)/*F*_m_′]; photochemical quenching coefficient [*qP* = (*F*_m_′ – *F*_s_)/(*F*_m_′ – *F*_0_′)]; non-photochemical quenching coefficient [*NPQ* = (*F*_m_ – *F*_m_′)/*F*_m_′].

### 2.10 Statistical analysis

Data were analyzed by one-way ANOVA using SPSS 20.0 software (SPSS Inc., Chicago, IL, USA). For data collected across multiple time points, statistical comparisons were conducted in two ways: (1) Differences among the three trellis systems at each individual time point; (2) Differences across time points within each trellis system. In all cases, Duncan’s multiple range test was used for *post-hoc* comparisons at a significance level of *p* < 0.05. Prior to ANOVA, the assumptions of the model were evaluated. Homogeneity of variances was confirmed using Levene’s test (p > 0.05). Due to the limited number of biological replicates (n = 3), which reduces the statistical power of formal normality tests, the assumption of normality was assessed graphically by examining normal quantile-quantile (Q-Q) plots of the residuals (Blanca et al., 2017). As the data showed no severe deviations from normality and variances were homogeneous, ANOVA was considered robust and appropriate for analysis even for this sample size (Schmider et al., 2010). Correlation analysis and principal component analysis (PCA) were conducted on physiological parameters collected on Day 15. SigmaPlot 14.0 (Systat Software Inc., San Jose, CA, USA) generated graphical presentations. Correlation heatmaps and PCA plots were generated using the online platform Chiplot (https://www.chiplot.online/, accessed 11 April 2025).

## 3 Results

### 3.1 Canopy temperature differences among trellis systems during summer high-temperature periods

**Figure 3:**
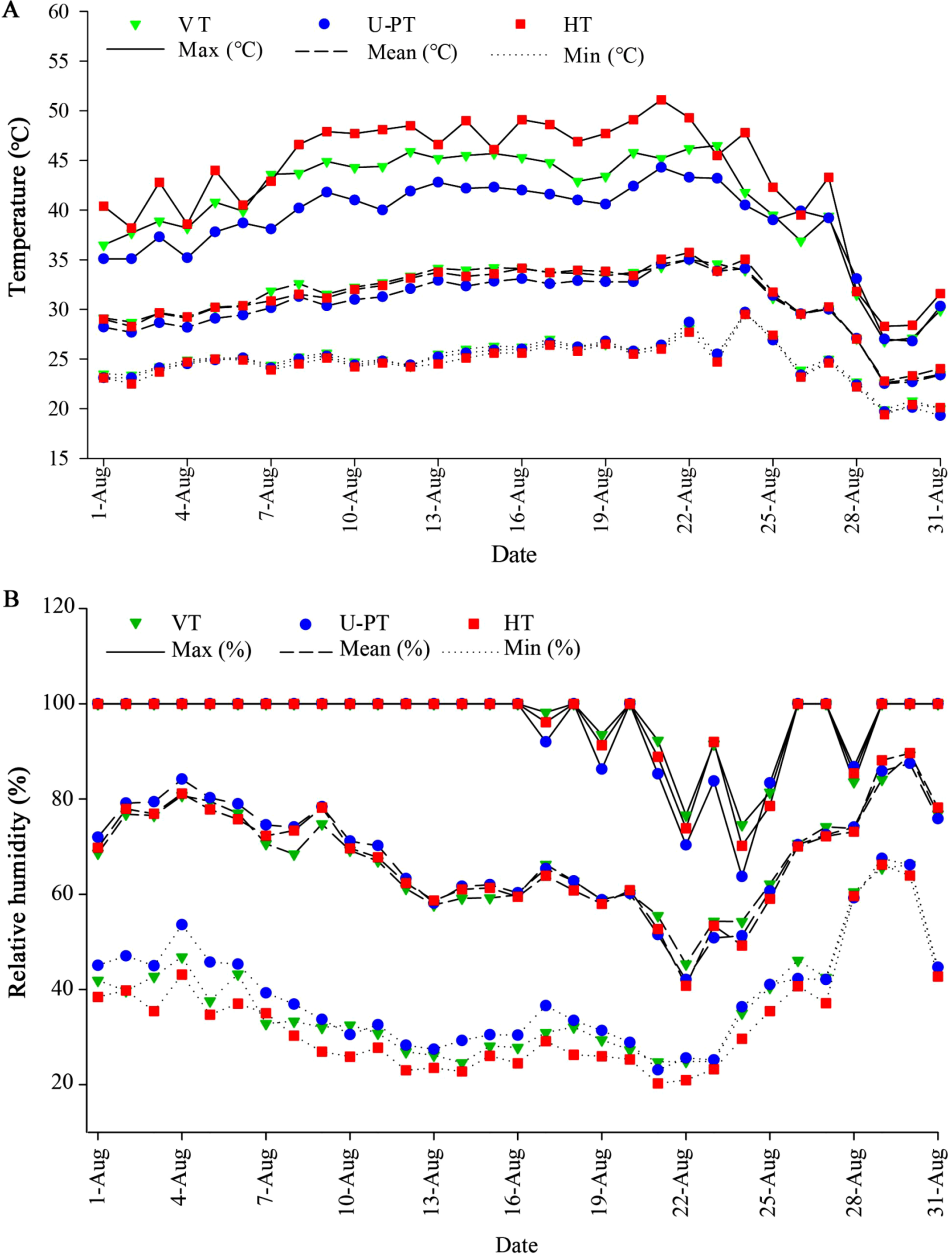
Daily mean, minimum, and maximum temperature **(A)** and relative humidity **(B)** within grape canopies under three trellis systems in August. Values indicate the means of three replicates.

Temperature monitoring during August 2022 (**Figure 3A**) showed that U-PT maintained more stable canopy temperatures with lower daily averages compared to other trellis systems. As detailed in **Table 1**, HT reached a maximum canopy temperature of 48.87°C, significantly exceeding U-PT by 3.84°C (*p* < 0.05). No significant difference in maximum temperature was observed between VT and U-PT (*p* > 0.05). Regarding cumulative heat exposure, HT and VT canopies endured ≥40°C temperatures for 14.07% and 13.78% of monitoring time respectively, equivalent to 1.86 and 1.82 times that of U-PT (7.57%) (*p* < 0.05 for both). More critically, durations ≥45°C reached 2.46% (HT) and 1.48% (VT), which were 18.92 and 11.38 times that of U-PT (0.13%) (*p* < 0.05 for both).

**Table 1:** Effect of trellis system on canopy temperature and high-temperature duration in August.

Trellis system	Mean temperature (°C)	Minimum temperature (°C)	Maximum temperature (°C)	Proportion of time ≥40 °C (%)	Proportion of time ≥45 °C (%)
VT	31.19 ± 0.13 a	19.90 ± 0.16 a	46.50 ± 1.06 ab	13.78 ± 0.42 b	1.48 ± 0.00 b
U-PT	30.46 ± 0.24 b	19.50 ± 0.15 b	45.03 ± 0.75 b	7.57 ± 1.13 c	0.13 ± 0.18 c
HT	31.28 ± 0.04 a	19.57 ± 0.21 ab	48.87 ± 2.20 a	14.07 ± 0.61 a	2.46 ± 0.62 a

Values indicate the means ± standard deviation (SD, n=3). Different lowercase letters indicate significant differences between trellis systems (one-way ANOVA, Duncan’s test, *p* < 0.05).

Further analysis of canopy RH (**Figure 3B**), conducted in August 2022, revealed a gradual decrease in humidity levels across the three trellis systems as the high-temperature stress progressed, followed by a gradual recovery after Day 15 (22 August) as canopy temperatures decreased. As detailed in **Table 2**, U-PT maintained the highest mean RH (68.31%), which was significantly higher than that of HT (67.54%) (*p* < 0.05), while VT showed an intermediate value (67.58%). In terms of humidity distribution, both U-PT and VT recorded a comparable percentage of time within the 60–80% RH range (approximately 25%) (*p* > 0.05), which was significantly longer than that of HT (20.68%) (*p* < 0.05 for both). Additionally, U-PT registered the highest proportion of time under >80% RH conditions (38.82%), significantly exceeding both VT and HT (approximately 36.4%) (*p* < 0.05 for both).

**Table 2:** Effect of trellis system on canopy relative humidity and high-temperature duration in August.

Trellis system	Mean RH (%)	Minimum RH (%)	Maximum RH (%)	Proportion of time at 60-80% RH (%)	Proportion of time >80% RH (%)
VT	67.58 ± 0.56 ab	24.60 ± 1.19 a	100.00 ± 0.00 a	25.20 ± 1.18 a	36.36 ± 0.85 b
U-PT	68.31 ± 0.31 a	23.10 ± 0.52 a	100.00 ± 0.00 a	24.93 ± 0.24 a	38.82 ± 0.30 a
HT	67.54 ± 0.38 b	20.27 ± 1.95 b	100.00 ± 0.00 a	20.68 ± 0.47 b	36.38 ± 0.80 b

Values indicate the means ± standard deviation (SD, n=3). Different lowercase letters indicate significant differences between trellis systems (one-way ANOVA, Duncan’s test, *p* < 0.05). RH, relative humidity.

### 3.2 Differential sunburn damage in grape leaves under three trellis systems

**Figure 4:**
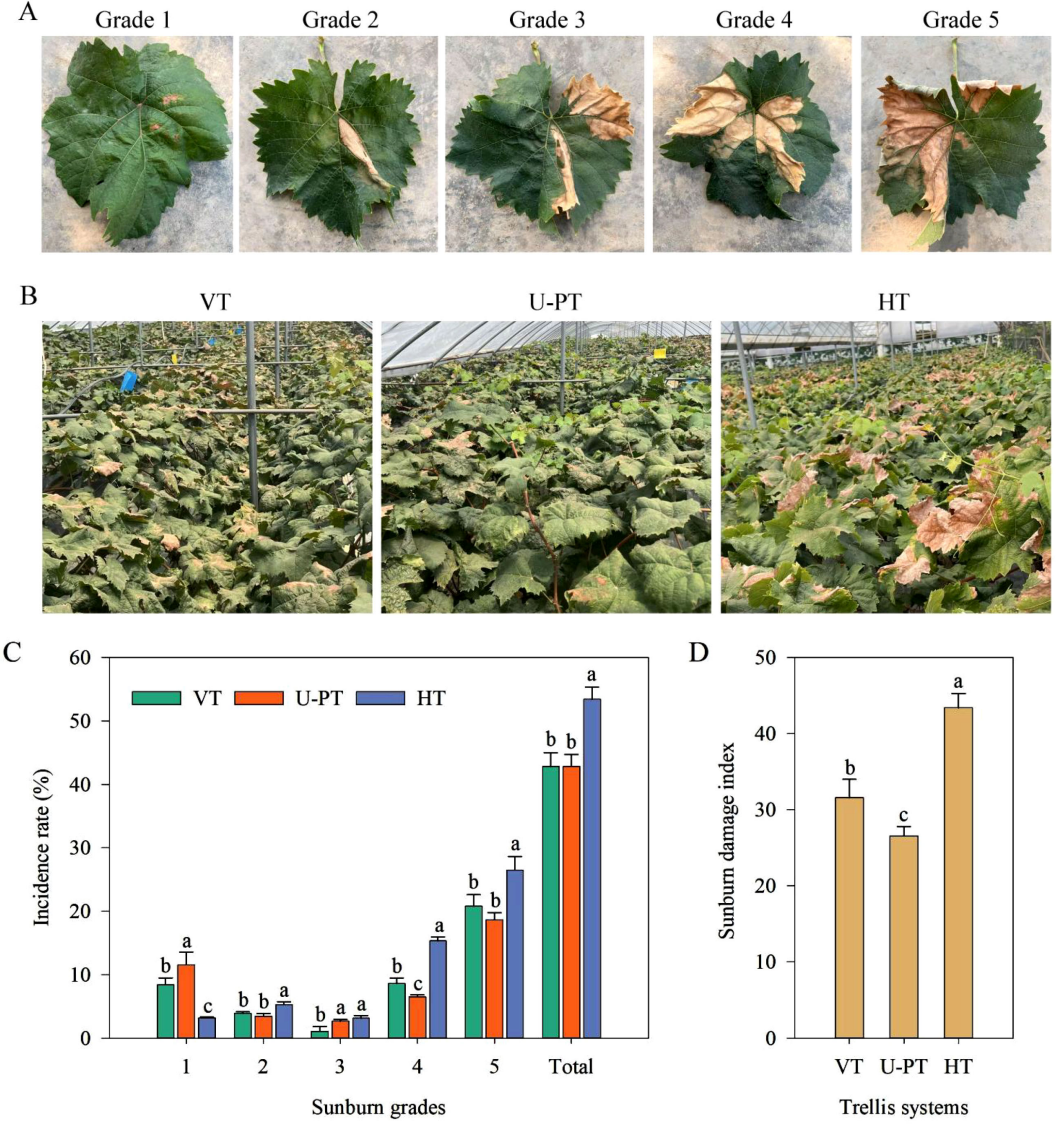
Sunburn characteristics in grapevine leaves under three trellis systems under prolonged summer heatwaves. **(A)** Leaf symptoms of different sunburn damage grades; **(B)** Canopy-scale sunburn manifestation; **(C)** Incidence rate distribution across sunburn grades and total occurrence; **(D)** Sunburn damage index. Values indicate the means of three replicates; error bars indicate the standard deviations. Different lowercase letters indicate significant differences between trellis systems (one-way ANOVA, Duncan’s test, *p* < 0.05). The sunburn damage index was calculated from a six-grade scale (0-5) of thermal necrosis area percentage (0% to ≥50%) to reflect the overall leaf damage severity.

After 15 consecutive days of prolonged high-temperature stress (**Figures 4A, B**), all trellis systems showed leaf sunburn damage with distinct severity levels. Both VT and HT exhibited a higher number of sunburn-affected leaves with larger scorched areas. In contrast, U-PT retained fewer damaged leaves, mainly showing tip and margin scorching and curling.

Quantitative analysis (**Figure 4C**) confirmed HT had the highest sunburn incidence (53.45% leaves affected), significantly surpassing VT (42.82%) and U-PT (42.84%) (*p* < 0.05 for both). Severe damage (Grade 5) predominated in VT (20.80%) and HT (26.49%), followed by Grade 4 (8.61%, 15.33% respectively), with <15% low-grade injuries. U-PT displayed a bimodal distribution: 18.67% Grade 5 and 11.56% Grade 1. The sunburn damage index (**Figure 4D**) demonstrated U-PT’s superiority with the lowest value (26.57), showing 15.86% and 38.76% reductions compared to VT and HT respectively (*p* < 0.05 for both).

### 3.3 Divergent stomatal responses to heat stress among trellis systems

**Figure 5:**
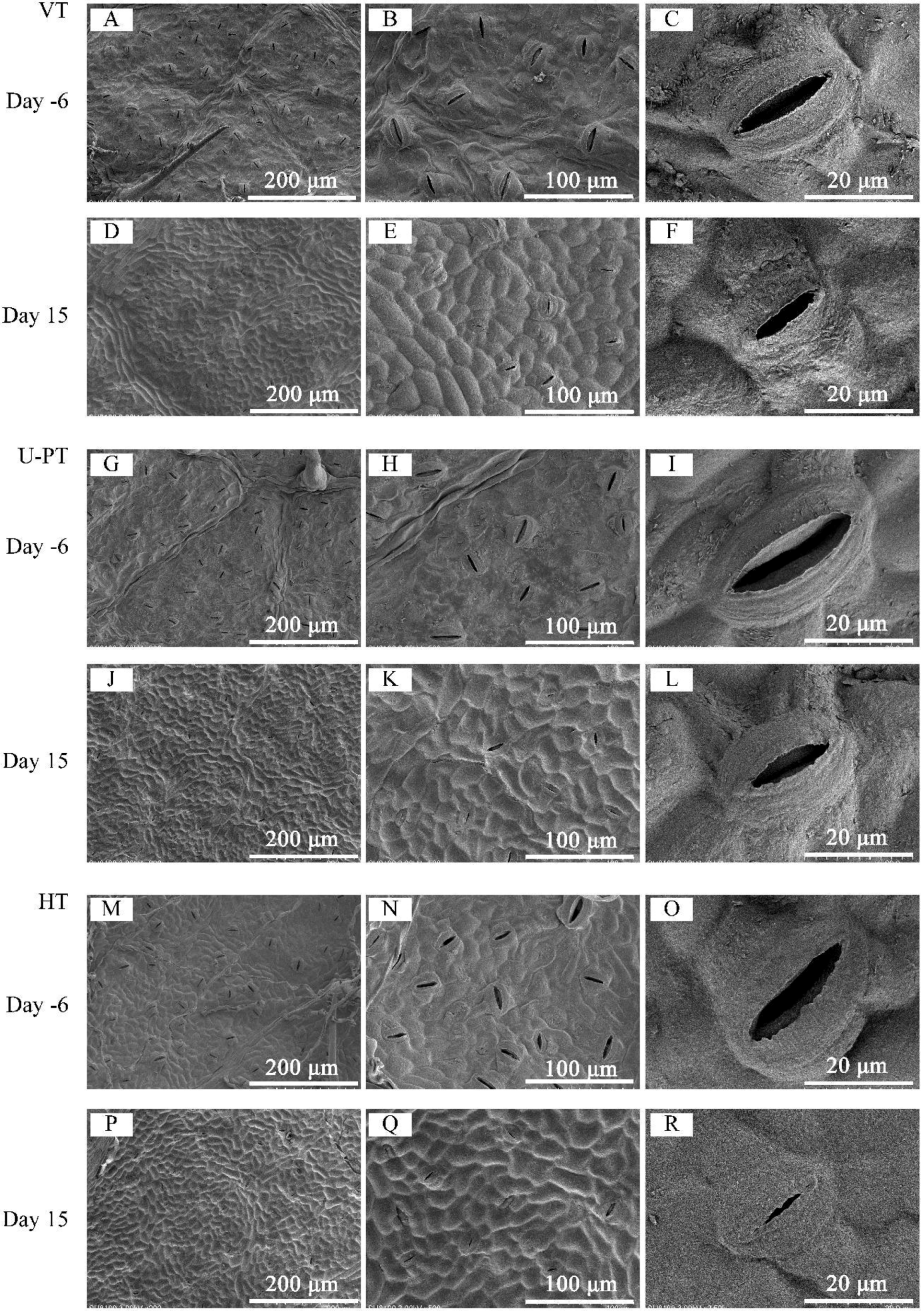
Effects of prolonged summer heatwaves on stomatal morphology in grapevine leaves under three trellis systems. **(A–C)** VT on Day -6; **(D–F)** VT on Day 15; **(G–I)** U-PT on Day -6; **(J–L)** U-PT on Day 15; **(M–O)** HT on Day -6; **(P–R)** HT on Day 15. Day -6 denotes the 6^th^ day prior to the onset of the high-temperature period, and Day 15 denotes the 15^th^ day of Prolonged high-temperature stress. Columns from left to right show micrographs at increasing magnifications: Column 1: General view of the leaf epidermis (200× magnification; scale bar = 200 µm), showing the density and distribution of stomata across the epidermal surface; Column 2: Morphology of stomatal complexes (500× magnification; scale bar = 100 µm), showing the arrangement of guard cells and their surrounding epidermal cells; Column 3: High-resolution detail of a single stoma, highlighting the guard cells and aperture (2500× magnification; scale bar = 20 µm).

**Figure 5** demonstrates significant morphological alterations in leaf stomata across three trellis systems following 15 consecutive days of high-temperature stress. Pre-stress observations revealed stomata protruding above the epidermis with turgid guard cells and expanded apertures (**Figure 5**, Day -6). Post-stress analysis showed stomata flush with the epidermis or sunken beneath it, accompanied by collapsed guard cells and substantially reduced aperture dimensions (**Figure 5**, Day 15 d). Epidermal anticlinal cell walls became more pronounced under heat stress.

As detailed in **Table 3**, all measured parameters (stomatal width, aperture length, aperture width, and aperture rate) exhibited significant reductions in all trellis systems after heat stress (*p* < 0.05), except for stomatal length in HT. Compared with their pre-stress values, VT, U-PT, and HT demonstrated respective decreases of 70.79%, 68.77%, and 76.80% in aperture length, with aperture width reductions reaching 79.91%, 76.02%, and 78.94%. Corresponding aperture rate declines measured 43.28%, 33.39%, and 45.17% respectively. Furthermore, U-PT maintained 16.25% and 21.09% higher aperture rates than VT and HT (*p* < 0.05 for both) respectively after heat stress. Notably, stomatal density remained unchanged relative to pre-stress levels (*p* > 0.05), with no statistical differences observed between trellis systems (*p* > 0.05), suggesting stomatal density may not be a key regulator in short-term heat adaptation.

**Table 3:** Stomatal responses to prolonged summer heatwaves in grapevine leaves across three trellis systems.

Trellis system	Sampling day	Stomatal length (μm)	Stomatal width (μm)	Stomatal aperture length (μm)	Stomatal aperture width (μm)	Stomatal aperture rate (%)	Stomatal density (no. mm^-2^)
VT	Day -6	31.84 ± 1.33 aA	20.52 ± 0.60 aA	21.33 ± 1.38 aA	4.28 ± 1.01 bA	98.83 ± 0.83 aA	165.38 ± 11.05 aA
	Day 15	23.24 ± 1.76 bB	16.40 ± 0.78 aB	6.23 ± 0.87 aB	0.86 ± 0.38 aB	56.06 ± 1.33 bB	170.52 ± 25.63 aA
U-PT	Day -6	32.80 ± 0.73 aA	22.32 ± 1.27 aA	23.12 ± 0.74 aA	6.88 ± 0.36 aA	97.84 ± 0.40 aA	188.45 ± 21.98 aA
	Day 15	26.44 ± 0.53 aB	17.62 ± 1.28 aB	7.22 ± 1.99 aB	1.65 ± 0.44 aB	65.17 ± 2.35 aB	178.81 ± 28.22 aA
HT	Day -6	26.52 ± 1.59 bB	16.88 ± 0.78 bA	20.52 ± 0.64 aA	5.84 ± 1.27 abA	98.16 ± 1.12 bA	158.84 ± 20.71 aA
	Day 15	25.52 ± 0.64 abB	13.52 ± 0.33 bB	4.76 ± 1.75 aB	1.23 ± 0.65 aB	53.82 ± 2.70 bB	132.49 ± 24.90 aA

Values indicate the means ± standard deviation (SD, n=3). Within a single time point, different lowercase letters indicate significant differences among the three trellis systems (one-way ANOVA, Duncan’s test, *p* < 0.05). Within a single trellis system, different uppercase letters indicate significant differences between the two sampling days (one-way ANOVA, Duncan’s test, *p* < 0.05). Sampling Time: Day -6 denotes the 6^th^ day prior to the onset of the high-temperature period, and Day 15 denotes the 15^th^ day of Prolonged high-temperature stress.

### 3.4 Divergent chloroplast ultrastructure responses to heat stress among trellis systems

**Figure 6:**
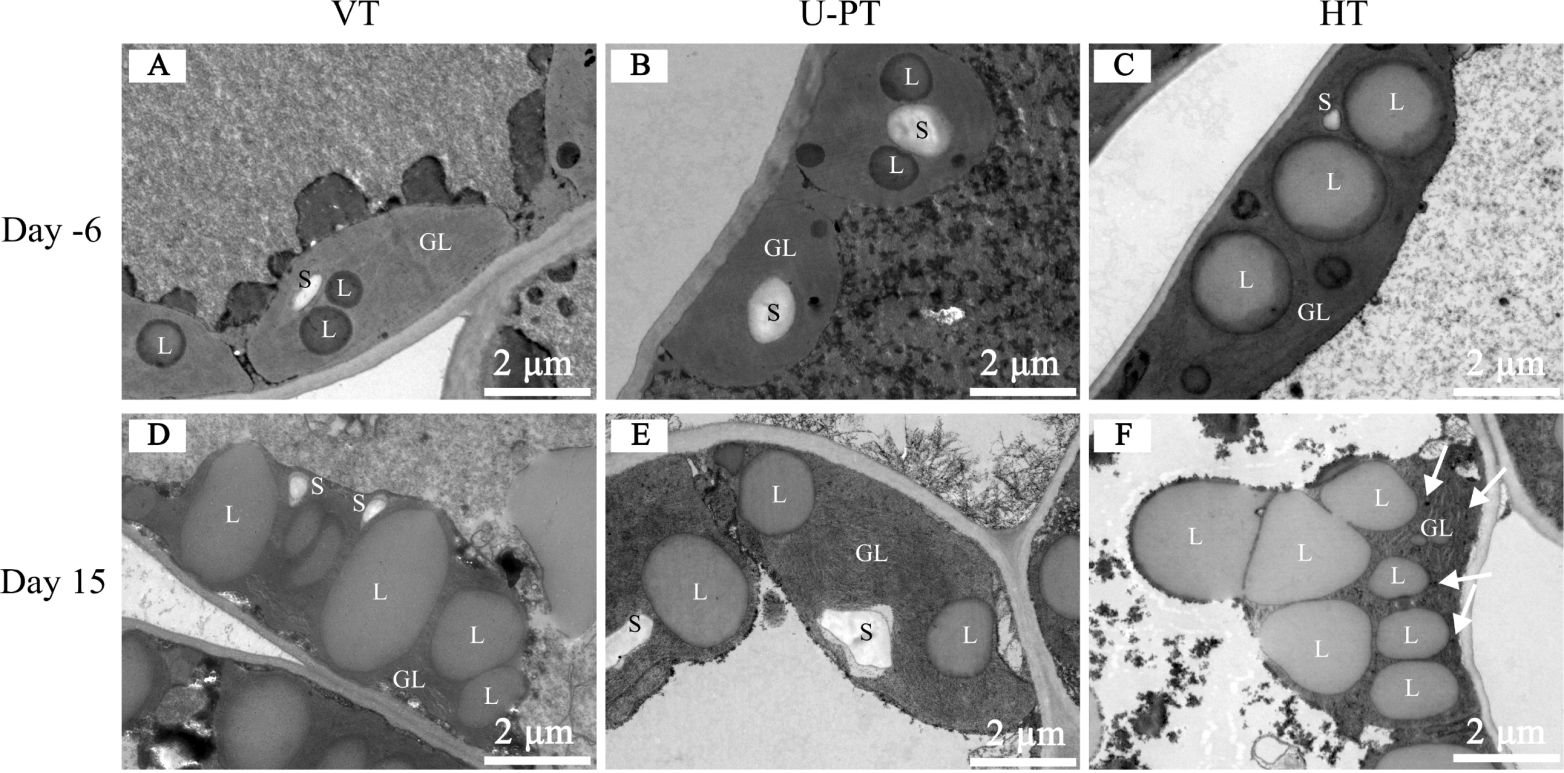
Effects of prolonged summer heatwaves on chloroplast ultrastructure in grapevine leaves under three trellis systems. **(A)** VT on Day -6; **(B)** U-PT on Day -6; **(C)** HT on Day -6; **(D)** VT on Day 15; **(E)** U-PT on Day 15; **(F)** HT on Day 15. Day -6 denotes the 6^th^ day prior to the onset of the high-temperature period, and Day 15 denotes the 15^th^ day of Prolonged high-temperature stress. S, Starch granule; L, lipid droplet; The white arrow in **(F)** indicates osmiophilic granules.

Pre-stress observations revealed that all three trellis systems exhibited normal chloroplast architecture (**Figure 6**). The ellipsoidal chloroplasts adhered to cell walls and contained starch granules (S) and lipid droplets (L, osmiophilic granule aggregates) in the stroma. HT chloroplasts displayed notably larger lipid droplets and smaller starch granules compared to VT and U-PT even before stress imposition. As detailed in **Table 4**, the length of lipid droplets in HT was significantly greater than that in VT and U-PT by 58.59% and 80.46%, respectively, while the width was significantly greater by 39.56% and 58.76%, respectively.

**Table 4:** Chloroplast and lipid droplet responses to prolonged summer heatwaves in grapevine leaves across three trellis systems.

Trellis system	Sampling day	Chloroplast		Lipid droplet		
		length (μm)	width (μm)	No./chloroplast	length (μm)	width (μm)
VT	Day -6	5.14 ± 0.97 bB	2.33 ± 0.29 aB	1.95 ± 0.90 aB	0.99 ± 0.06 bB	0.91 ± 0.09 bB
	Day 15	8.97 ± 0.74 aA	5.01 ± 0.56 bA	4.83 ± 2.20 aA	1.66 ± 0.15 abA	1.27 ± 0.30 bA
U-PT	Day -6	5.10 ± 0.63 bB	2.20 ± 0.45 aB	1.81± 1.45 aA	0.87 ± 0.09 bB	0.80 ± 0.10 bB
	Day 15	7.05 ± 0.83 bA	3.86 ± 0.65 cA	2.40 ± 0.80 bA	1.47 ± 0.16 bA	1.23 ± 0.29 bA
HT	Day -6	8.47 ± 0.60 aA	3.19 ± 0.56 aB	2.63 ± 1.33 aB	1.57 ± 0.58 aB	1.27 ± 0.25 a B
	Day 15	8.67 ± 0.70 aA	7.54 ± 0.25 aA	6.44 ± 1.94 aA	1.87 ± 0.34 aA	1.72 ± 0.55 aA

Values indicate the means ± standard deviation (SD, n=3). Within a single time point, different lowercase letters indicate significant differences among the three trellis systems (one-way ANOVA, Duncan’s test, *p* < 0.05). Within a single trellis system, different uppercase letters indicate significant differences between the two sampling days (one-way ANOVA, Duncan’s test, *p* < 0.05). Sampling Time: Day -6 denotes the 6^th^ day prior to the onset of the high-temperature period, and Day 15 denotes the 15^th^ day of Prolonged high-temperature stress.

Following 15 consecutive days of high-temperature stress, distinct ultrastructural responses emerged (**Figure 6**). VT and HT chloroplasts showed marked expansion with lipid droplets occupying most stromal space. HT chloroplasts progressed to spherical morphology with envelope integrity loss. and accompanied by complete starch granule depletion. Quantitative data (**Table 4**) on Day 15 showed that chloroplast length in VT and HT was significantly greater than in U-PT by 27.23% and 22.98%, respectively, and chloroplast width was significantly greater by 29.79% and 95.34%, respectively. The number of lipid droplets per chloroplast in VT and HT was significantly higher than in U-PT by 101.25% and 168.33%, respectively. Regarding lipid droplet size, no significant differences were observed between VT and U-PT. However, HT exhibited significantly larger lipid droplets than both VT and U-PT, with length greater by 12.65% and 27.21%, and width greater by 35.43% and 39.84%, respectively. In summary, in contrast to VT and HT, U-PT chloroplasts exhibited minimal volumetric expansion, alongside substantially smaller lipid droplets and larger-sized starch granules, indicating better structural integrity under prolonged heat stress.

### 3.5 Dynamics of chlorophyll a, chlorophyll b, and total chlorophyll during summer high-temperature periods

**Figure 7:**
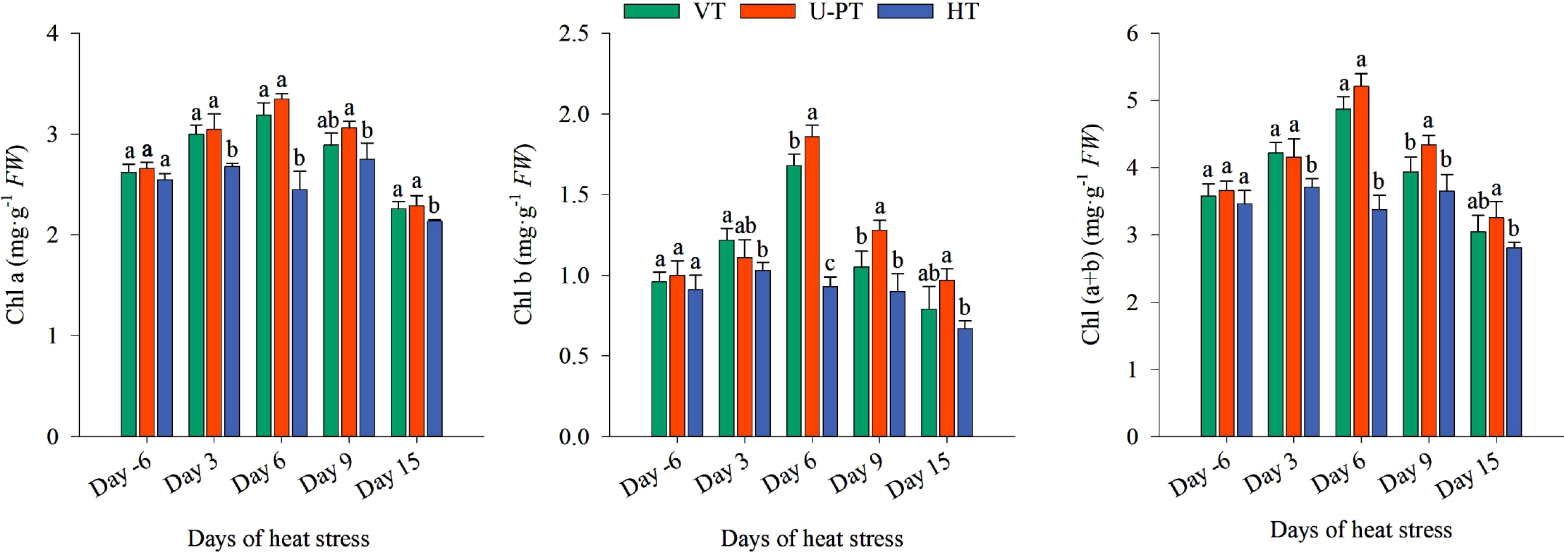
Effects of prolonged summer heatwaves on chlorophyll content in grapevine leaves under three trellis systems. Day -6 denotes the 6^th^ day prior to the onset of the high-temperature period, Day 3, Day 6, Day 9 and Day 15 denote the 3^rd^, 6^th^, 9^th^, and 15^th^ day of Prolonged high-temperature stress, respectively. Values indicate the means of three replicates; error bars indicate the standard deviations. Different lowercase letters at the same time point indicate significant differences between trellis systems (one-way ANOVA, Duncan’s test, *p* <no><</no> 0.05). Chl a, Chlorophyll a content; Chl b, Chlorophyll b content; Chl (a+b), Chlorophyll (a+b) content.

No significant differences in chlorophyll content were observed among trellis systems at the pre-stress timepoint (Day -6) (*p* > 0.05; **Figure 7**). During summer high-temperature periods, Chl a and Chl b contents initially increased then declined in all trellis systems. VT and U-PT reached peak contents of Chl a and Chl b on Day 6, while HT peaked earlier on Day 3. From Day 6, chlorophyll retention ranked as U-PT > VT > HT. By Day 15, total chlorophyll decreased by 10.93% (U-PT), 14.80% (VT), and 18.79% (HT) relative to pre-stress levels. U-PT maintained 16.01% higher total chlorophyll than HT (*p* < 0.05), with VT showing intermediate values that were not significantly different from either U-PT or HT (*p* > 0.05 for both).

### 3.6 Photosynthetic parameters dynamics during summer high-temperature periods

**Figure 8:**
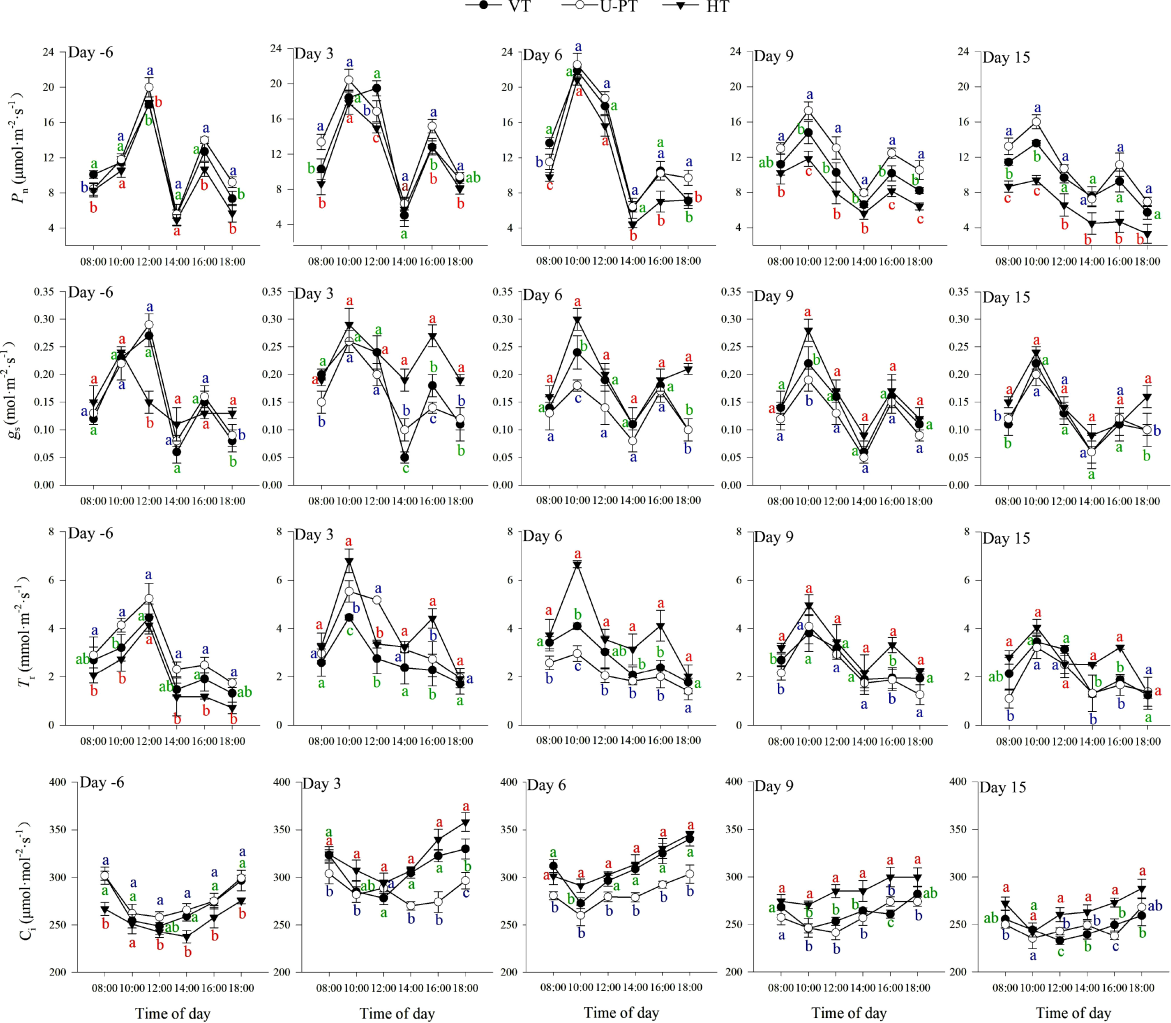
Diurnal dynamics of photosynthetic parameters in three grapevine trellis systems during summer high-temperature periods. Day -6 denotes the 6^th^ day prior to the onset of the high-temperature period, Day 3, Day 6, Day 9 and Day 15 denote the 3^rd^, 6^th^, 9^th^, and 15^th^ day of Prolonged high-temperature stress, respectively. Values indicate the means of three replicates; error bars indicate the standard deviations. Different lowercase letters at the same time point indicate significant differences between trellis systems with colors corresponding to each system: green (VT), blue (U-PT), and red (HT) (one-way ANOVA, Duncan’s test, *p* < 0.05). *P*_n_, net photosynthetic rate; *g*_s_, stomatal conductance; *T*_r_, transpiration rate; *C*_i_, intercellular CO_2_ concentration.

As shown in **Figure 8**, the diurnal patterns of *P*_n_, *G*_S_, and *T*_r_ were consistent across the three trellis systems, forming bimodal curves—with two exceptions: *G*_S_ in HT exhibited a “rise-fall-rise” trend from 8:00 to 18:00 on Day -6, Day 6, and Day 15; *T*_r_ in VT and HT showed a transient increase followed by a decrease on Day 3. On Day -6, the highest peaks of *P*_n_, *g*_s_, and *T*_r_ generally occurred at 12:00 for all trellis systems, except for *G*_S_ in HT leaves, which peaked at 10:00. From Day 3 to Day 15, the diurnal highest peaks advanced to 10:00 for all three parameters. Throughout the observation period, secondary peaks generally occurred at 16:00, while troughs occurred at 14:00. *C*_i_ in all trellis systems followed a declining-then-increasing diurnal trend, with minimum values at 12:00–14:00 from Day -6 to Day 3 and at 10:00–12:00 from Day 6 to Day 15.

From Day -6 to Day 15, *P*_n_, *g*_s_, *T*_r_, and *C*_i_ across all three trellis systems exhibited a trend of increasing first and then decreasing, peaking on Day 3 or Day 6. During high-temperature periods, *P*_n_ followed the order U-PT > VT > HT, with differences between trellis systems progressively widening over time. By Day 15, the average *P*_n_ from 8:00 to 18:00 of U-PT, VT, and HT had decreased by 5.47%, 12.25%, and 36.42% respectively, relative to pre-stress levels. Additionally, the average *P*_n_ of U-PT was 14.21% and 76.22% higher than that of VT and HT, respectively (*p* < 0.05 for both). On Day 3 and Day 6, the average *g*_s_, *T*_r_, and *C*_i_ differed significantly among trellis systems, with values following HT > VT > U-PT (*p* < 0.05 for all comparisons). These disparities diminished over time: by Day 15, *g*_s_ and *T*_r_ showed no significant differences between U-PT, VT, and HT (*p* > 0.05 for all comparisons), except that the average *C*_i_ of HT was significantly higher than those of VT and U-PT (*p* < 0.05 for both).

### 3.7 WUEi dynamics during summer high-temperature periods

**Figure 9:**
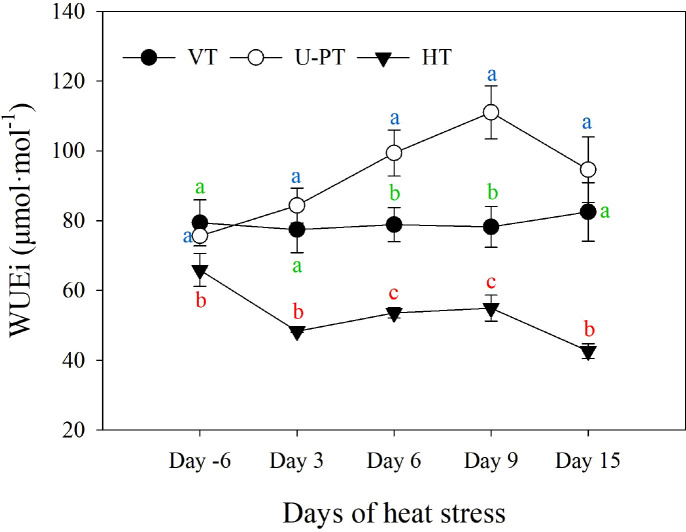
Effects of prolonged summer heatwaves on intrinsic water-use efficiency (WUEi) of grapevine leaves under three trellis systems. Day -6 denotes the 6^th^ day prior to the onset of the high-temperature period, Day 3, Day 6, Day 9 and Day 15 denote the 3^rd^, 6^th^, 9^th^, and 15^th^ day of Prolonged high-temperature stress, respectively. Values indicate the means of three replicates; error bars indicate the standard deviations. Different lowercase letters at the same time point indicate significant differences between trellis systems with colors corresponding to each system: green (VT), blue (U-PT), and red (HT) (one-way ANOVA, Duncan’s test, *p* < 0.05).

Throughout the prolonged high-temperature stress period, the WUEi of leaves under the three trellis systems exhibited distinct dynamics (**Figure 9**). The WUEi of HT showed an overall decreasing trend, and by Day 15, it had decreased by 35.25% compared to the pre-stress level. In contrast, the WUEi of VT remained relatively stable over the course of the stress period. The WUEi of U-PT increased initially and then declined, peaking on Day 9 with an increase of 46.75% compared to the pre-stress level. By Day 15, it remained 25.06% higher than the initial value. Throughout the stress period, the WUEi values across the three trellis systems consistently followed the order: U-PT > VT > HT. On Day 15, there was no significant difference between U-PT and VT, (*p* > 0.05) but both were significantly higher than HT (*p* < 0.05 for both), with increases of 121.96% and 93.57%, respectively.

### 3.8 chlorophyll fluorescence parameter dynamics during summer high-temperature periods

**Figure 10:**
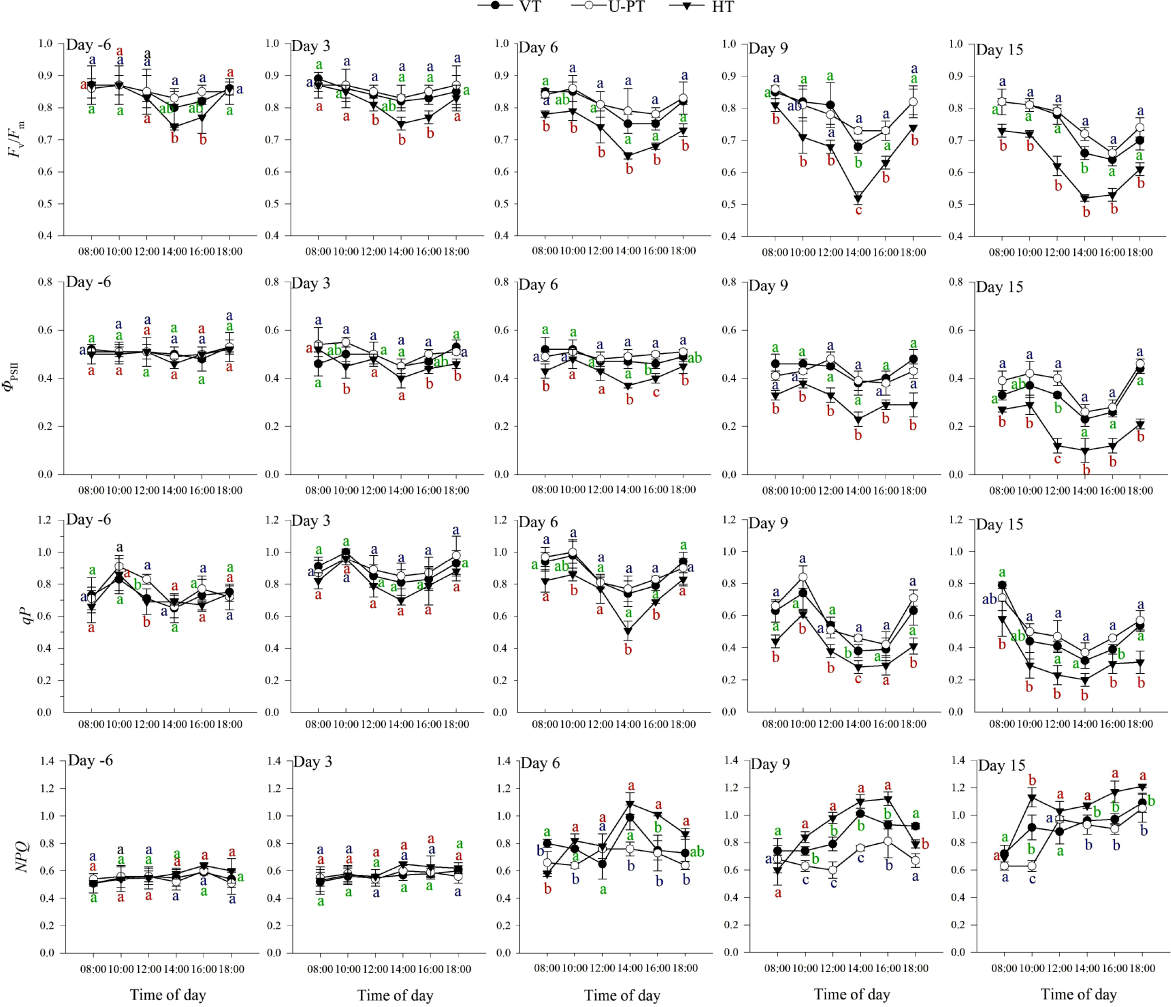
Diurnal dynamics of chlorophyll fluorescence parameters in three grapevine trellis systems during summer high-temperature periods. Day -6 denotes the 6^th^ day prior to the onset of the high-temperature period, Day 3, Day 6, Day 9 and Day 15 denote the 3^rd^, 6^th^, 9^th^, and 15^th^ day of Prolonged high-temperature stress, respectively. Values indicate the means of three replicates; error bars indicate the standard deviations. Different lowercase letters at the same time point indicate significant differences between trellis systems with colors corresponding to each system: green (VT), blue (U-PT), and red (HT) (one-way ANOVA, Duncan’s test, *p* < 0.05). *F*_v_/*F*_m_, maximum quantum yield of PSII; *Φ*_PSII_, effective quantum yield of PSII; *qP*, photochemical quenching coefficient; *NPQ*, non-photochemical quenching coefficient.

As shown in **Figure 10**, *F*_v_/*F*_m_ and *Φ*_PSII_ exhibited minor diurnal variations across all trellis systems on Day -6 and Day 3, with only slight midday declines. As stress duration lengthened, both parameters across all trellis systems gradually declined: *F*_v_/*F*_m_ developed a pronounced V-shaped diurnal pattern, reaching a minimum at 14:00, while *Φ*_PSII_ followed a “rise-fall-rise” pattern, peaking at 10:00 before declining to a trough at 14:00—a pattern consistent with *qP* diurnal variations observed from Day -6 to Day 9. By Day 15, *qP* transitioned to showing a morning maximum followed by a continuous decline to a 14:00 minimum. Notably, *qP* values transiently increased on Day 3 compared to pre-stress levels but declined thereafter as stress duration lengthened.

From Day -6 to Day 9, *NPQ* diurnal curves across all trellis systems exhibited an initial increase followed by a decrease, peaking between 14:00 and 16:00. However, by Day 15, *NPQ* trajectories shifted to a sustained upward trend. During the initial high-temperature stress (Day -6 to Day 3), the diurnal amplitudes of *NPQ* were relatively small. From Day 6, both the diurnal amplitudes and absolute values of *NPQ* increased progressively.

Comparative analysis identified HT as the most sensitive to heat stress. By Day 15, HT displayed significantly lower daytime-averaged *F*_v_/*F*_m_ (0.62), *Φ*_PSII_ (0.19), and *qP* (0.32) compared to VT (0.73, 0.33, 0.48) and U-PT (0.76, 0.37, 0.52) (*p* < 0.05 for all comparisons). Conversely, HT’s average *NPQ* (1.05) exceeded both VT (0.92) and U-PT (0.85) (*p* < 0.05 for both). No significant differences in chlorophyll fluorescence parameters were observed between VT and U-PT (*p* > 0.05), except for *NPQ* (*p* < 0.05).

### 3.9 Correlation analysis

**Figure 11:**
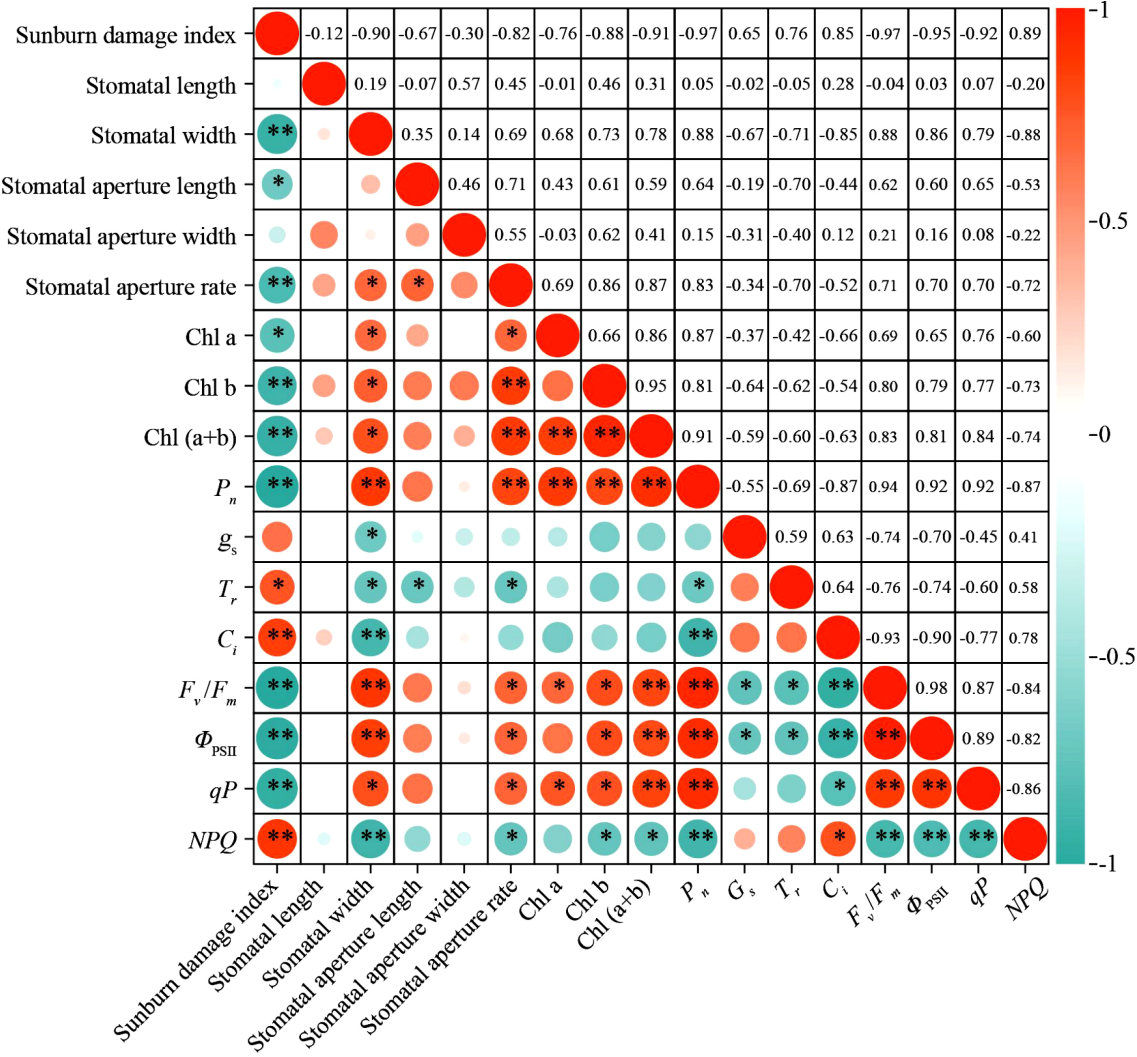
Pearson correlations among indices related to leaf sunburn damage, stomatal traits, photosynthesis, and chlorophyll fluorescence under prolonged summer heatwaves. Asterisks indicate significance levels (**p < 0.01, *p < 0.05). Circle size and color intensity reflect correlation strength, with red indicating positive correlations and cyan denoting negative correlations. Values are Pearson correlation coefficients. Chl a, Chlorophyll a content; Chl b, Chlorophyll b content; Chl (a+b), Chlorophyll (a+b) content; *P*_n_, net photosynthetic rate; *g*_s_, stomatal conductance; *T*_r_, transpiration rate; *C*_i_, intercellular CO_2_ concentration; *F*_v_/*F*_m_, maximum quantum yield of PSII; *Φ*_PSII_, effective quantum yield of PSII; *qP*, photochemical quenching coefficient; *NPQ*, non-photochemical quenching coefficient.

Pearson correlation analysis was conducted to evaluate relationships among 17 indicators, including leaf sunburn damage index, stomatal morphological traits, chlorophyll content, photosynthetic parameters, and chlorophyll fluorescence parameters (**Figure 11**). The results revealed positive correlations among five variables: sunburn damage index, *g*_s_, *T*_r_, *C*_i_, and *NPQ*. Conversely, these five variables exhibited negative correlations with 12 other parameters, such as stomatal length, stomatal aperture ratio, chlorophyll content [Chl a, Chl b, and Chl (a+b)], *P*_n_, *F*_v_/*F*_m_, *Φ*_PSII_, and *qP*, while the latter group showed mutual positive correlations. Notably, *F*_v_/*F*_m_ exhibited significant (*p* < 0.05) to extremely significant (*p* < 0.01) correlations with stomatal aperture, chlorophyll content, and photosynthetic and chlorophyll fluorescence parameters. This confirms that *F*_v_/*F*_m_ can reflect plant heat tolerance by integrating information on stomatal status and photosystem stability.

### 3.10 Principal component analysis (PCA) among the three trellis systems

**Figure 12:**
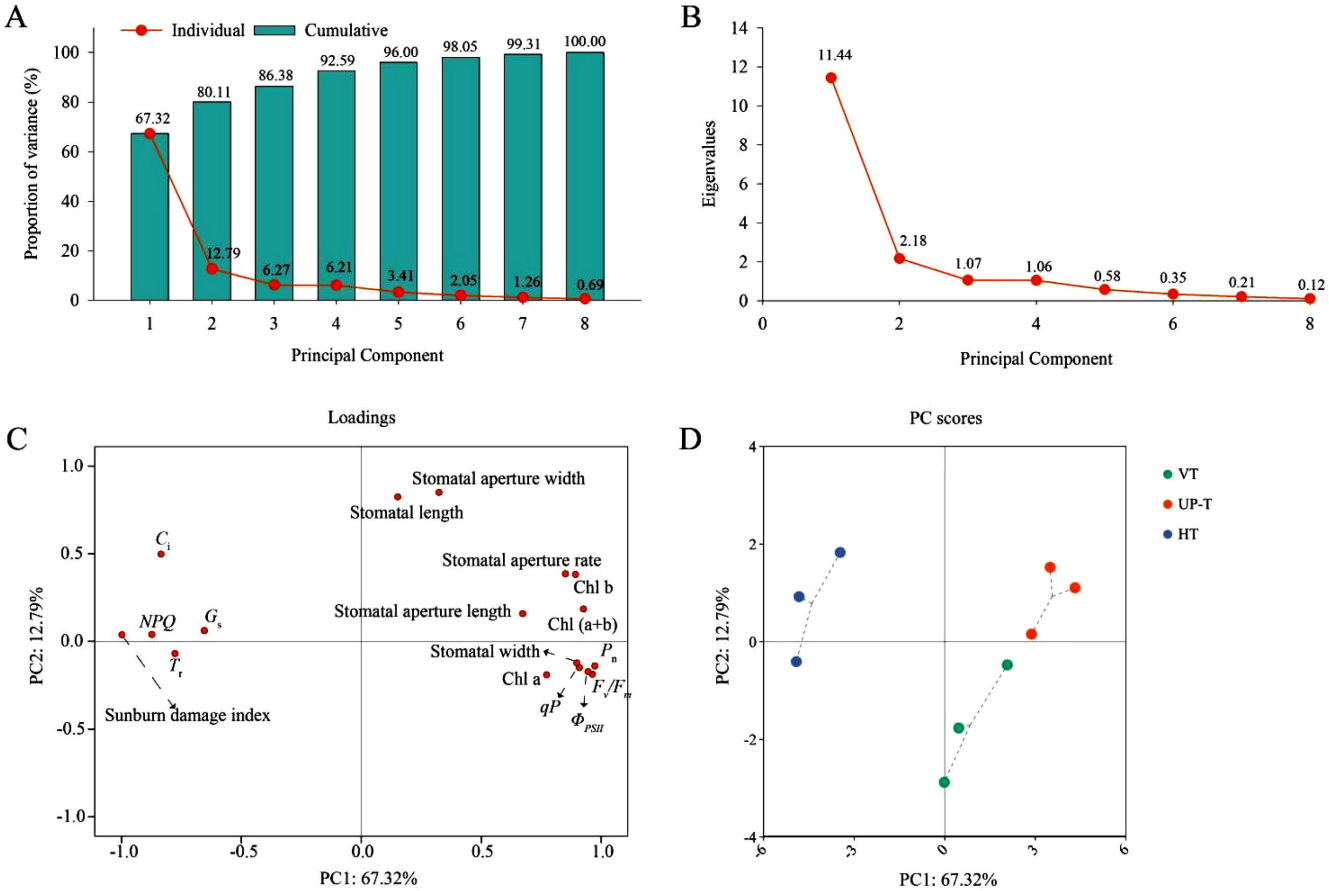
PCA analysis of the three grapevine trellis systems under prolonged summer heatwaves. **(A)** Variance of the principal components (PCs); **(B)** Eigenvalues of the PCs; **(C)** Loading plot of variables; **(D)** PCA score plot. PC1, the first principal component; and PC2, the second principal component. Chl a, Chlorophyll a content; Chl b, Chlorophyll b content; Chl (a+b), Chlorophyll (a+b) content; *P*_n_, net photosynthetic rate; *g*_s_, stomatal conductance; *T*_r_, transpiration rate; *C*_i_, intercellular CO_2_ concentration; *F*_v_/*F*_m_, maximum quantum yield of PSII; *Φ*_PSII_, effective quantum yield of PSII; *qP*, photochemical quenching coefficient; *NPQ*, non-photochemical quenching coefficient.

Principal component analysis (PCA) was conducted to comprehensively evaluate the heat tolerance of different grape trellis systems under high-temperature stress, using the 17 indicators from the previous correlation analysis. The first four principal components (PCs) collectively accounted for 92.59% of the total variance (eigenvalues: 11.44, 2.18, 1.07, and 1.06) (**Figures 12A, B**). Notably, the first two PCs (PC1 and PC2) explained 67.32% and 12.79% of the variance respectively, with a cumulative contribution rate of 80.11% (**Figure 12A**), indicating their sufficiency in explaining dataset variability. In PC1, the sunburn damage index, *g*_s_, *T*_r_, *C*_i_, and *NPQ* exhibited negative loadings (λ), whereas the remaining 12 variables showed positive loadings (**Figure 12C**). Among these, the sunburn damage index, *P*_n_, *F*_v_/*F*_m_, *Φ*_PSII_, Chl (a+b), and *qP* contributed most significantly to PC1 (absolute λ > 0.9). In contrast to PC1, PC2 showed distinct loading patterns: stomatal width, Chl a, *P*_n_, *T*_r_, *F*_v_/*F*_m_, *Φ*_PSII_, and *qP* had negative loadings, while the remaining ten variables exhibited positive loadings (**Figure 12C**). Stomatal length and stomatal aperture width demonstrated the highest loadings on PC2 (λ > 0.8).

PCA-derived scores revealed distinct heat tolerance profiles among trellis systems. While U-PT and VT exhibited minimal separation in the PC score space, both diverged markedly from HT (**Figure 12D**). Following Peng et al. (2023) ‘s methodology, composite scores were calculated for each trellis system based on PC1 and PC2 (**Table 5**), where higher scores indicated superior heat tolerance. The heat tolerance ranking was: U-PT (1.82) > VT (0.94) > HT (-0.42), confirming U-PT’s enhanced capacity to maintain photosynthetic stability under high-temperature stress.

**Table 5:** Comprehensive evaluation of heat tolerance of the three grapevine trellis systems.

Trellis system	Principal component score		Comprehensive score	Ranking
	PC1	PC2		
VT	1.08	0.22	0.94	2
U-PT	1.97	1.05	1.82	1
HT	-0.70	1.07	-0.42	3

## 4 Discussion

### 4.1 Trellis system-dependent modulation of canopy temperature and humidity

Under the dual pressures of global warming, high-temperature stress in vineyards has emerged as a critical challenge for sustainable viticulture. Trellis systems, as the structural framework of grape cultivation, can significantly influence plant heat tolerance (Liu et al., 2015; Gladstone and Dokoozlian, 2003). This study investigated the influence of trellis systems on the heat tolerance of ‘Shine Muscat’ grapevines.

In the rain-shelter cultivation, the top film blocks the outward escape of long-wave radiation emitted by the soil surface and plants (Chase et al., 1999). Hot air rises and accumulates at the top of the shelter, and heat dissipation can only rely on slow horizontal gas exchange between the sides of the shelter (which remain uncovered, unlike the film-covered top) and the outside environment (Lim et al., 2017). This low heat dissipation efficiency further exacerbates high-temperature stress inside the shelter during summer.

Our study demonstrated that different trellis systems exhibited significant differences in canopy temperature during high-temperature periods. Among them, HT had the highest canopy temperature and the most severe leaf sunburn damage. Its horizontal canopy is not only inherently prone to absorbing solar radiation and thus heating up, but more critically, it exacerbates the inherent structural limitations of the rain-shelter: the dense horizontal canopy intercepts solar radiation transmitted through the top film, and this radiation absorption—coupled with the heat-retention effect of the top film—collectively induces severe heat accumulation above the canopy (Florence, 2017). Despite the restricted air movement, the extreme heat under HT likely elevated the saturation vapor pressure deficit, which can intensify transpirational water loss from leaves while simultaneously reducing the relative humidity of the canopy air mass. VT showed intermediate canopy temperatures between HT and U-PT. Its V-shaped canopy helps reduce solar radiation interception per unit leaf area at noon, and the open canopy structure facilitates vertical convection. However, due to its smaller canopy spread angle (60°) and lower trunk height (1.0 m), horizontal air circulation remains restricted (Reynolds and Vanden Heuvel, 2009), thus leading to its higher canopy temperature compared to U-PT.

U-PT exhibited superior microclimate regulation performance. It was characterized by the lowest daily mean canopy temperature, minimal temperature variability, and the highest mean relative humidity among the trellis systems. Moreover, it endured a markedly shorter cumulative duration of extreme high temperatures (≥45°C), accounting for only 5.28% and 8.78% of that in HT and VT, respectively. This advantage is attributable to its unique “high trunk-wide opening-pendulous shoot” architecture: the elevated trunk (1.5 m) and wide canopy opening angle (130°) significantly enhance both horizontal and vertical air circulation within the rain-shelter, while the pendulous new shoots effectively reduce direct solar radiation interception. Correspondingly, U-PT showed the lowest leaf sunburn damage index among all tested trellis systems. Collectively, U-PT confers significant advantages for rain-shelter cultivation, making it particularly well-suited for grape-growing regions with high heat accumulation risks.

### 4.2 Trellis system-dependent modulation of stomatal morphology under heat stress

Stomata, as specialized epidermal gas-exchange channels in plants (Hetherington and Woodward, 2003), directly regulate leaf photosynthetic-transpiration coupling efficiency through their size, aperture, density, and spatial distribution (Chang et al., 2023). After 15 consecutive days of high-temperature stress, significant reductions in stomatal width, aperture length, aperture width, and aperture ratio were observed across all three trellis systems, while stomatal density remained unchanged. Short-term heat exposure typically induces stomatal opening to enhance transpirational cooling (Santanoo et al., 2022), whereas prolonged heat stress triggers stomatal closure or aperture reduction to balance transpirational water loss and leaf temperature (Cheng et al., 2023; Sarwar et al., 2019). Divergent reports on high-temperature effects on stomatal density—ranging from no significant impact (Yang et al., 2024) to increases (Jumrani et al., 2016) or decreases (Yang et al., 2021)—likely reflect species-specific genetic responses and experimental variability. Stomatal number, size, and spatial distribution define the limits of physiological regulation—higher density and larger apertures generally indicate enhanced heat tolerance (Driesen et al., 2020; Haworth et al., 2021). Despite the absence of inter-trellis differences in stomatal density or aperture dimensions post-stress, U-PT exhibited a significantly higher stomatal aperture ratio than VT and HT (*p* < 0.05), demonstrating its superior capacity for stomatal regulation under prolonged heat stress.

### 4.3 Trellis system-dependent modulation of chlorophyll and chloroplast ultrastructure under heat stress

High-temperature stress exerts profound impacts on chloroplast structure and functionality. Excessive reactive oxygen species (ROS) accumulation under heat stress inhibits chlorophyll synthesis (Mustafa et al., 2021; Lim et al., 2007) and promotes its degradation (Mattila et al., 2018), accompanied by disintegration of the chloroplast envelope and thylakoid structures (Zhao et al., 2022), collectively driving chloroplast dysfunction. However, our study revealed transient increases in Chl a and Chl b contents during initial high-temperature stress across all trellis systems. We hypothesize that timely activation of antioxidant systems (such as SOD and APX) effectively scavenges early-stage ROS, delaying chlorophyll degradation while upregulating chlorophyll biosynthesis-related genes to compensate for photosystem damage-induced declines in light-harvesting efficiency. This phenomenon is consistent with chlorophyll dynamics observed in the herbaceous peony (*Paeonia lactiflora*) cultivars ‘Bo Baishao’ and ‘Fenyunu’ under natural high-temperature conditions (Wang et al., 2022).

Divergent responses emerged among trellis systems under prolonged heat stress. VT and U-PT maintained later chlorophyll content peaks (Day 6) with smaller subsequent reductions, whereas HT exhibited earlier peaking (Day 3) followed by rapid depletion, suggesting insufficient ROS scavenging efficiency leading to premature chlorophyll degradation in HT leaves (Wang et al., 2024). Chloroplast ultrastructure observations corroborated these findings: following 15 consecutive days of high-temperature stress, both VT and HT chloroplasts displayed abnormally swollen morphology, along with substantial lipid droplet accumulation—hallmark products of chloroplast membrane lipid peroxidation (Arzac et al., 2022; Jalil et al., 2023). Notably, HT also exhibited ruptured chloroplast envelopes. Prior to stress (Day -6), HT chloroplasts already showed larger lipid droplets and smaller starch granules compared to other systems (**Figure 3**), likely due to sufficiently high pre-stress canopy temperatures that caused early cellular damage. This precondition and the higher canopy temperature predisposed HT to more severe peroxidative damage upon subsequent heat exposure. In contrast, U-PT chloroplasts maintained structural integrity and significantly lower lipid droplet accumulation compared to other trellis systems.

### 4.4 Photosynthetic and chlorophyll fluorescence dynamics: from acclimation to photoinhibition

Our data showed that prolonged heat stress triggered consistent responses in photosynthetic parameters (*P*_n_, *g*_s_, *T*_r_, *C*_i_) across all trellis systems, mirroring chlorophyll content dynamics with initial upregulation followed by progressive decline. The transient photosynthetic enhancement during initial high-temperature stress likely stemmed from short-term acclimation mechanisms, including increased stomatal conductance to enhance transpirational cooling and transient chlorophyll biosynthesis activation. This pattern aligns with observations by Dou et al. (2021) conducted in five grape cultivars, which showed temporary increases in *P*_n_, *g*_s_, *T*_r_, and *C*_i_ under initial heat stress. However, prolonged high-temperature stress ultimately led to a reduction in photosynthetic rate during the later stages.

Under heat stress, the reduction in *P*_n_ can be attributed to both stomatal and non-stomatal limitations (Luis et al., 2016). In this study, reductions in *g*_s_ and *C*_i_ across all trellis systems indicated stomatal involvement in the *P*_n_ decline. However, severe chloroplast damage in VT and HT leaves suggested additional non-stomatal limitation, especially in HT. Analysis of WUEi further elucidated the predominant photosynthetic limitation in each system. The continuous decline in WUEi in HT indicated a greater reduction in *P*_n_ than in *g*_s_, confirming non-stomatal limitation as the primary constraint. In contrast, stable WUEi in VT reflected a proportional decline in *P*_n_ and *g*_s_, consistent with stomatal limitation. Notably, U-PT showed a substantial increase in WUEi, indicating maintained photosynthetic capacity under more efficient stomatal regulation. Thus, the *P*_n_ decline in U-PT likely resulted from a proactive stomatal strategy, protecting the photosynthetic machinery from severe heat damage.

Chlorophyll fluorescence analysis further validated these findings. *F*_v_/*F*_m_, a key probe for reflecting the degree of environmental stress, quantifies the potential maximum photochemical efficiency of the PSII reaction center (Doğru, 2021) and exhibits a positive correlation with plant heat tolerance (Wang et al., 2022), remaining stable under non-stress conditions (EI-Hendawy et al., 2019). *qP* represents the proportion of absorbed photons utilized for photochemical electron transport, while *NPQ* represents the proportion of absorbed photons dissipated as heat (Xu et al., 2020). In this study, *F*_v_/*F*_m_ and *NPQ* of three trellis systems remained stable during initial stress, indicating an intact PSII reaction center. Concurrently, *qP* increased, consistent with trends in chlorophyll content and *P*_n_. As heat stress continued, however, *NPQ* rose, suggesting plants mitigated photodamage by dissipating excess photons as heat—a regulatory pattern consistent with findings in lettuce seedlings (He et al., 2022) and squash seedlings (Qin et al., 2010). Across all trellis systems, *qP*, *F*_v_/*F*_m_, and *Φ*_PSII_ progressively declined in later stages of stress, indicating damage to the PSII reaction center and impairment of light energy conversion efficiency.

It should be noted that gas exchange measurements conducted under a standardized 25°C condition, an optimal temperature for photosynthesis (Uri et al., 2015), primarily reflect the potential photosynthetic performance of leaves after exposure to the distinct thermal environments induced by different trellis systems, rather than the *in-situ* photosynthetic rates of grapevines under actual field heat stress. The superior *P*_n_ and *g*_s_ values exhibited by U-PT under this controlled, standardized condition underscore its enhanced capacity to retain higher photosynthetic physiological potential following heat stress.

### 4.5 Potential mechanisms for enhanced heat tolerance in U-PT

In our study, under identical high-temperature stress durations, U-PT and VT maintained higher *F*_v_/*F*_m_, *Φ*_PSII_, *qP*, and *P*_n_ relative to HT. While U-PT showed no significant differences in *F*_v_/*F*_m_, *Φ*_PSII_, or *qP* compared to VT (*p* > 0.05), it exhibited a higher *P*_n_ (*p* < 0.05). The PCA confirmed U-PT as the most heat-tolerant trellis system.

These photosynthetic superiority may be attributed to enhanced intrinsic acclimation mechanisms. In our parallel study conducted under identical experimental conditions (Luo et al., 2025), we found that the activities of key antioxidant enzymes—superoxide dismutase (SOD), peroxidase (POD), and catalase (CAT)—in all three trellis systems showed an initial increase followed by a decrease. However, U-PT exhibited a delayed activity peak, and its overall enzyme activity was higher than that in the VT and HT throughout the stress period, which was consistent with higher expression levels of the corresponding genes (*VvSOD* and *VvCAT*). Consequently, U-PT accumulated significantly lower levels of ROS (O_2_^-^· and H_2_O_2_) and malondialdehyde (MDA), coupled with the smallest increase in relative electrical conductivity.

These findings indicate that the superior canopy architecture of U-PT mitigates heat stress by creating a milder and more stable thermal environment, thereby avoiding the overwhelming impact of extreme heat on cellular defense systems that occurs in HT. This favorable microclimate allows the antioxidant system in U-PT to be effectively activated without being rapidly depleted, ensuring continuous scavenging of excess ROS and protecting photosynthetic apparatus (such as chloroplast integrity and the PSII complex) from oxidative damage, which ultimately contributes to the maintenance of higher photosynthetic efficiency in U-PT.

### 4.6 Study limitations and future perspectives

The results of this study are only applicable to ‘Shine Muscat’ grapevines under rain-shelter cultivation, and their applicability to other grape varieties, regional climates, or cultivation patterns requires further verification. Furthermore, the expression of chlorophyll content on a fresh weight basis is acknowledged as a limitation. It should be noted that the trellis systems represent a holistic treatment that concurrently affects vine vigor, water relations, and canopy microclimate; our study evaluated their integrated performance under heat stress. Future work should employ targeted experiments to dissect the contribution of these individual factors. Molecular biology techniques can be used to explore the molecular mechanisms underlying the enhanced heat tolerance of grapevines by U-PT. Long-term field experiments should be conducted to evaluate the effects of different trellis systems on grape yield and fruit quality, so as to clarify the economic feasibility of their application in production. Meanwhile, canopy structure manipulation experiments or microclimate simulation can be employed to verify the hypothesis of canopy heat accumulation in HT, thereby providing theoretical support for the optimization of trellis systems.

## 5 Conclusion

This study addresses the knowledge gap regarding how trellis systems influence heat tolerance in ‘Shine Muscat’ Our findings establish that trellis system is a critical determinant of canopy temperature, leaf microstructure, and photosynthetic efficiency under prolonged heat stress. During prolonged summer high-temperature periods, the chlorophyll content and photosynthetic parameters (*P*_n_, *g*_s_, *T*_r_, *C*_i_) of ‘Shine Muscat’ initially showed transient increases, likely reflecting the plant’s short-term acclimation strategy to heat stress. As heat stress continued, photosynthetic efficiency gradually declined due to reduced stomatal aperture, disruption of chloroplast structure, chlorophyll degradation, and impaired PSII reaction center. Compared to VT and HT, U-PT maintained the lowest intensity and shortest duration of high canopy temperatures along with higher canopy RH, exhibited the minimal leaf sunburn damage index, and sustained the highest stomatal aperture, PSII reaction center activity, chlorophyll content, and most stable chloroplast structure—thereby preserving superior photosynthetic efficiency under heat stress. HT exhibited the poorest performance. The PCA confirmed that U-PT was the most heat-tolerant among the tested trellis systems. These findings provide a theoretical foundation for selecting heat-resistant trellis systems and investigating heat tolerance mechanisms in viticulture.

## Data Availability

The original contributions presented in the study are included in the article/**Supplementary Material**. Further inquiries can be directed to the corresponding authors.
