# Supplementary material for: Trellis systems ameliorate heat damage by regulating canopy temperature, photosynthetic efficiency and leaf microstructure of grapevine
Source: Front Plant Sci. 2026 Feb 4;16:1648999. doi: 10.3389/fpls.2025.1648999 (PMC12914953; doi:10.3389/fpls.2025.1648999)
Supplement: Supplementary file 1 [file DataSheet1.docx]

# Supplementary materials

This section provides detailed protocols for the uniform pruning, flower and fruit management, water-fertilizer regime, and pest and disease control practices applied to all three trellis systems (VT, U-PT, and HT) in this study.

**Pruning.** Shoot thinning and positioning were carried out after budbreak to maintain a shoot density of 10 to 14 shoots per square meter and an inter-shoot spacing of 20 cm. Winter pruning was performed primarily in the form of spur pruning, leaving 2- to 3-bud spurs.

**Irrigation strategy.** Irrigation **was applied** based on soil moisture status, phenological stage, and vine vigor. Adequate soil moisture (70–80% of field capacity) was maintained during budbreak, berry development, post-harvest, and pre-dormancy periods. Irrigation was restricted during the period around flowering and the ripening stage, **with light irrigation applied only** when soil moisture dropped below 40%.

****Fertilizer application.** I**n late October, basal fertilizer was applied in trenches 40–50 cm from the trunk, comprising 21,000 kg/ha of well-decomposed organic fertilizer, 330 kg/ha of balanced compound fertilizer (N–P₂O₅–K₂O, 15–15–15), and 525 kg/ha of calcium superphosphate. During the growing season, drip fertigation was applied as follows: before budbreak, 150 kg/ha of balanced compound fertilizer plus 45 kg/ha of urea; within 10 days after petal fall, 375 kg/ha of high-potassium compound fertilizer; at veraison (the onset of fruit softening), 375 kg/ha of potassium sulfate plus 150 kg/ha of calcium superphosphate; within 7 days after harvest, 75 kg/ha of balanced compound fertilizer plus 45 kg/ha of borax. Foliar fertilization was applied as follows: 0.2% borax once 7–14 days before flowering; 0.2%–0.3% monopotassium phosphate at 7–10 day intervals from berry development to veraison (3–4 applications total); 3% calcium superphosphate during the second **fruit expansion** phase. Micronutrient and secondary nutrient fertilizers were supplemented as needed.

**Pest and Disease Control.** During the grape dormant period, a whole-vineyard spray of lime sulfur (3–5°Bé) was applied. One week before budbreak, lime sulfur (3–5°Bé) was sprayed again across the vineyard. Throughout the growing season, Bordeaux mixture (1:0.5:200) was used for integrated disease prevention. Diseases and pests were monitored closely, and highly effective, low-toxicity, low-residue chemical pesticides were applied promptly upon detection.

**Flower and fruit management.** Cluster thinning commenced at inflorescence emergence, with retention criteria based on shoot vigor: one cluster was kept on moderate shoots, up to two on strong shoots, and none on weak shoots. The ratio of fruiting shoots to vegetative shoots was maintained at 4:1. Cluster trimming were further carried out to regulate the total vineyard yield to approximately 22,500 kg/ha.
